# Supplementary material for: A C. elegans model of C9orf72-associated ALS/FTD uncovers a conserved role for eIF2D in RAN translation
Source: Nat Commun. 2021 Oct 15;12:6025. doi: 10.1038/s41467-021-26303-x (PMC8519953; doi:10.1038/s41467-021-26303-x)

SUPPLEMENTARY INFORMATION

Supplementary Figures 1 – 31
Supplementary Table 1
Supplementary File 1

A *C. elegans* model of *C9orf72*-associated ALS/FTD uncovers a conserved role for eIF2D in RAN translation

Yoshifumi Sonobe [1, 2, 3], Jihad Aburas [1, 3, 4], Gopinath Krishnan [5], Andrew C Fleming [6], Ghanashyam Ghadge [1, 2, 3], Priota Islam [7, 8], Eleanor C Warren [7, 8], Yuanzheng Gu [9], Mark W. Kankel [9], André E.X. Brown [7, 8], Evangelos Kiskinis [6], Tania F. Gendron [10], Fen-Biao Gao [5], Raymond P. Roos* [1, 2, 3], Paschalis Kratsios* [1, 3, 4]

* These authors contributed equally to this study.

Correspondence:

R.P.R (rroos@neurology.bsd.uchicago.edu), P.K (pkratsios@uchicago.edu)

Affiliations:

[1] University of Chicago Medical Center, 5841 S. Maryland Ave., Chicago, IL 60637

[2] Department of Neurology, University of Chicago Medical Center, 5841 S. Maryland Ave., Chicago, IL 60637

[3] The Grossman Institute for Neuroscience, Quantitative Biology, and Human Behavior, University of Chicago, Chicago, IL, USA

[4] Department of Neurobiology, University of Chicago, Chicago, IL, USA

[5] Department of Neurology, University of Massachusetts Medical School, Worcester, MA 01605, USA

[6] The Ken & Ruth Davee Department of Neurology, Feinberg School of Medicine, Northwestern University, Chicago, USA

[7] MRC London Institute of Medical Sciences, London, UK

[8] Institute of Clinical Sciences, Imperial College London, London, UK

[9] Neuromuscular & Movement Disorders, Biogen, Cambridge, MA 02142, USA

[10] Department of Neuroscience, Mayo Clinic, Jacksonville, FL, USA

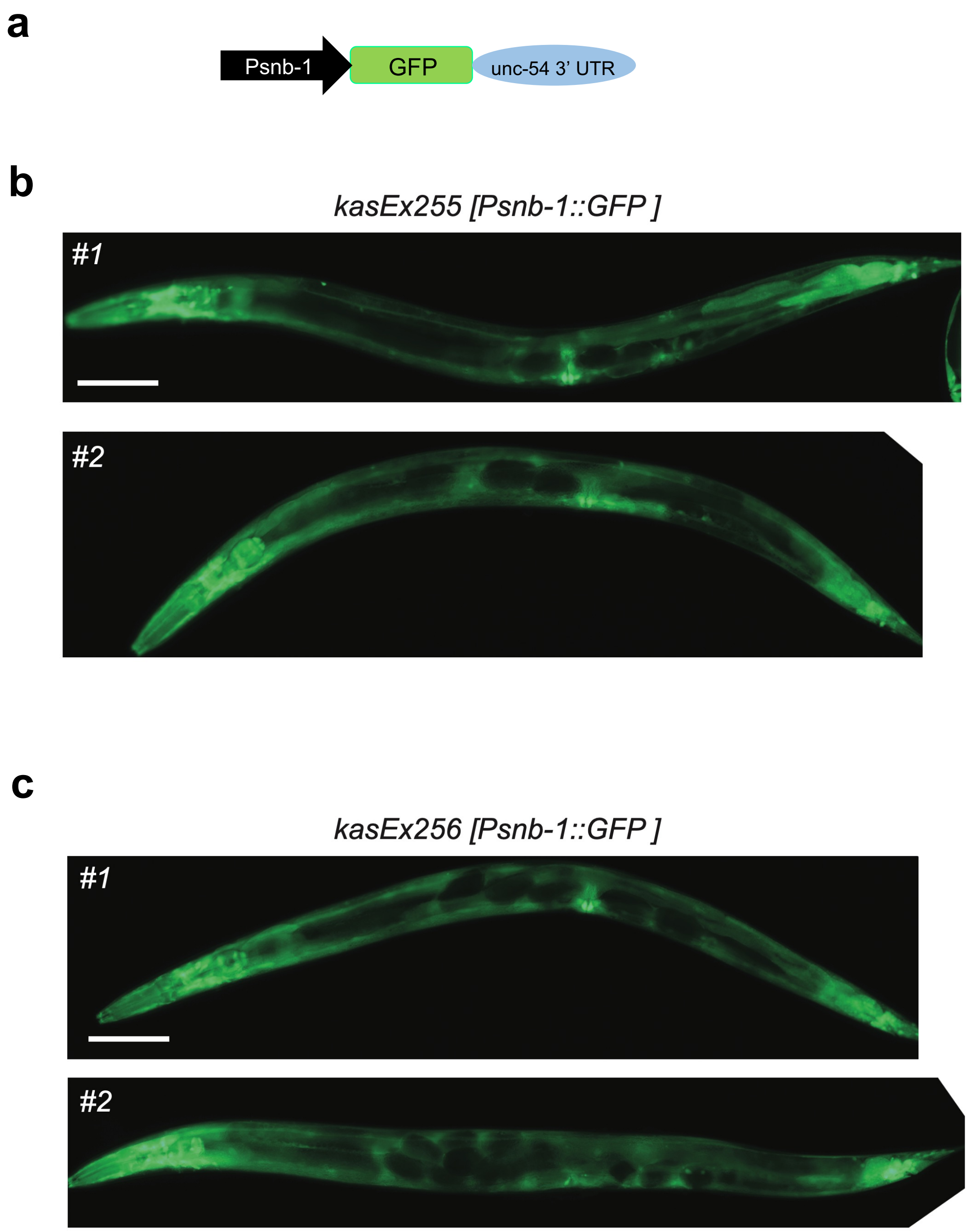

Supplementary Figure 1: The *snb-1* promoter drives broad GFP expression in *C. elegans*. (a) Schematic of construct used in panels b and c. GFP: green fluorescent protein; UTR: untranslated region.
(b) Representative images of two young adult (day 1) animals carrying the *kasEx255 [Psnb-1_1094bp::GFPnovo2+unc-54 3’UTR]* transgene. **(c)** Similar widespread GFP expression was observed with an independent transgenic line *kasEx256 [Psnb-1_1094bp::GFPnovo2+unc-54 3’UTR].* Ten animals per line were imaged. No GFP expression is observed in the intestinal lumen and the embryos (oval shapes) inside the body of these hermaphrodite animals. Scale bar = 50μm.

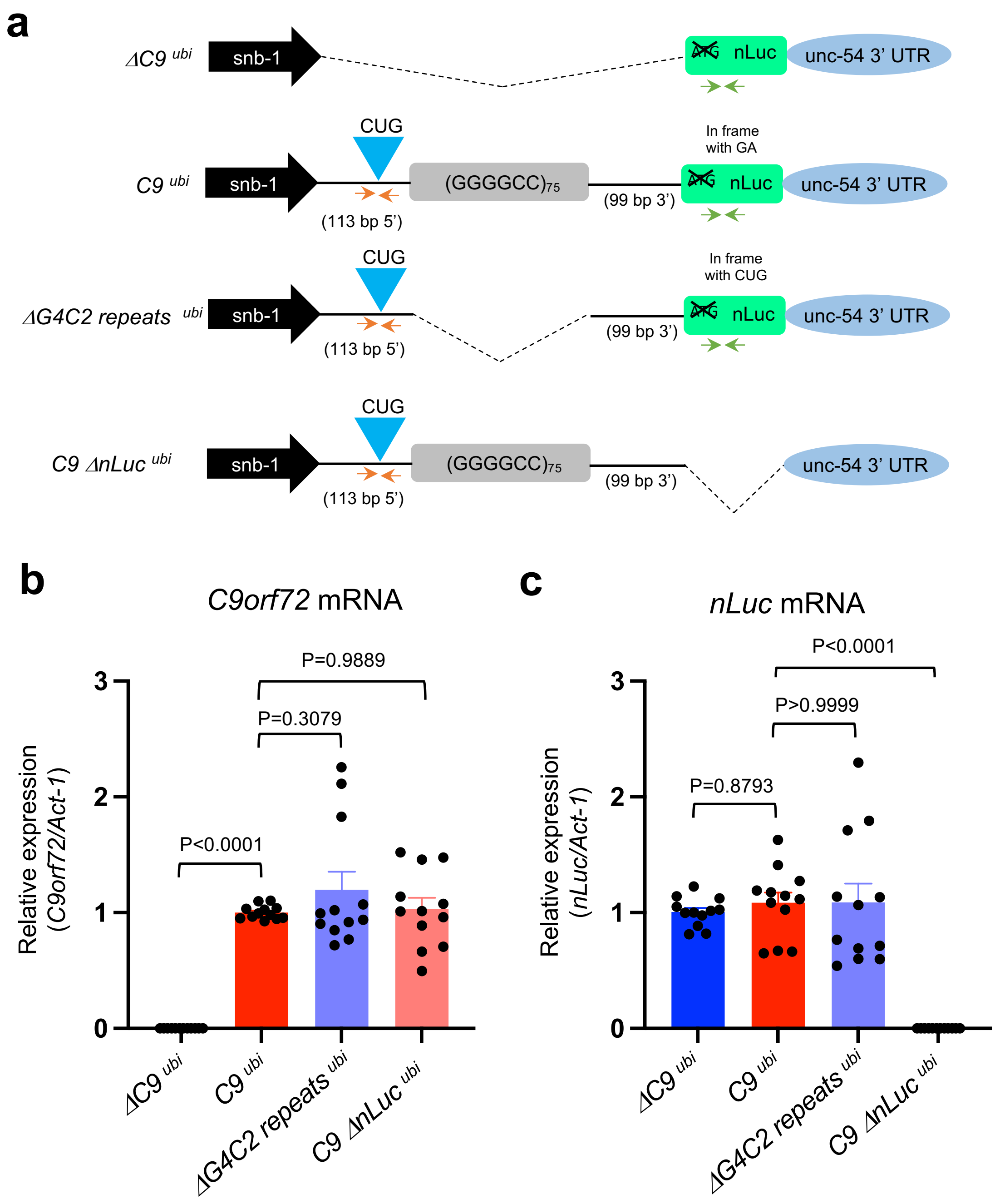

Supplementary figure 2. Expression levels of *C9orf72* and *nLuc* mRNA in ΔC9 *ubi*, C9 *ubi*, ΔG4C2 repeat *ubi*, and C9 (ΔnLuc) *ubi* worms. (a) Schematic diagram showing *ΔC9 ubi (KasEx154), C9 ubi (KasEx153), ΔG4C2 repeat ubi (KasEx240)*, and *C9 ΔnLuc ubi (KasEx237)* constructs. The orange and green arrows primer location to detect intronic *C9orf72* and nLuc mRNAs, respectively. nLuc: nanoluciferase; UTR: untranslated region. (b) The G4C2 repeat RNA (with surrounding *C9orf72* intronic RNA) and Act-1 mRNAs were assessed by RT-PCR. (c) The *nLuc* and Act-1 mRNAs were assessed by RT-PCR. The experiments used for (b) and (c) were repeated four times. The mean ± s.e.m. is shown. One-way ANOVA with Dunnett's multiple comparisons test was performed.

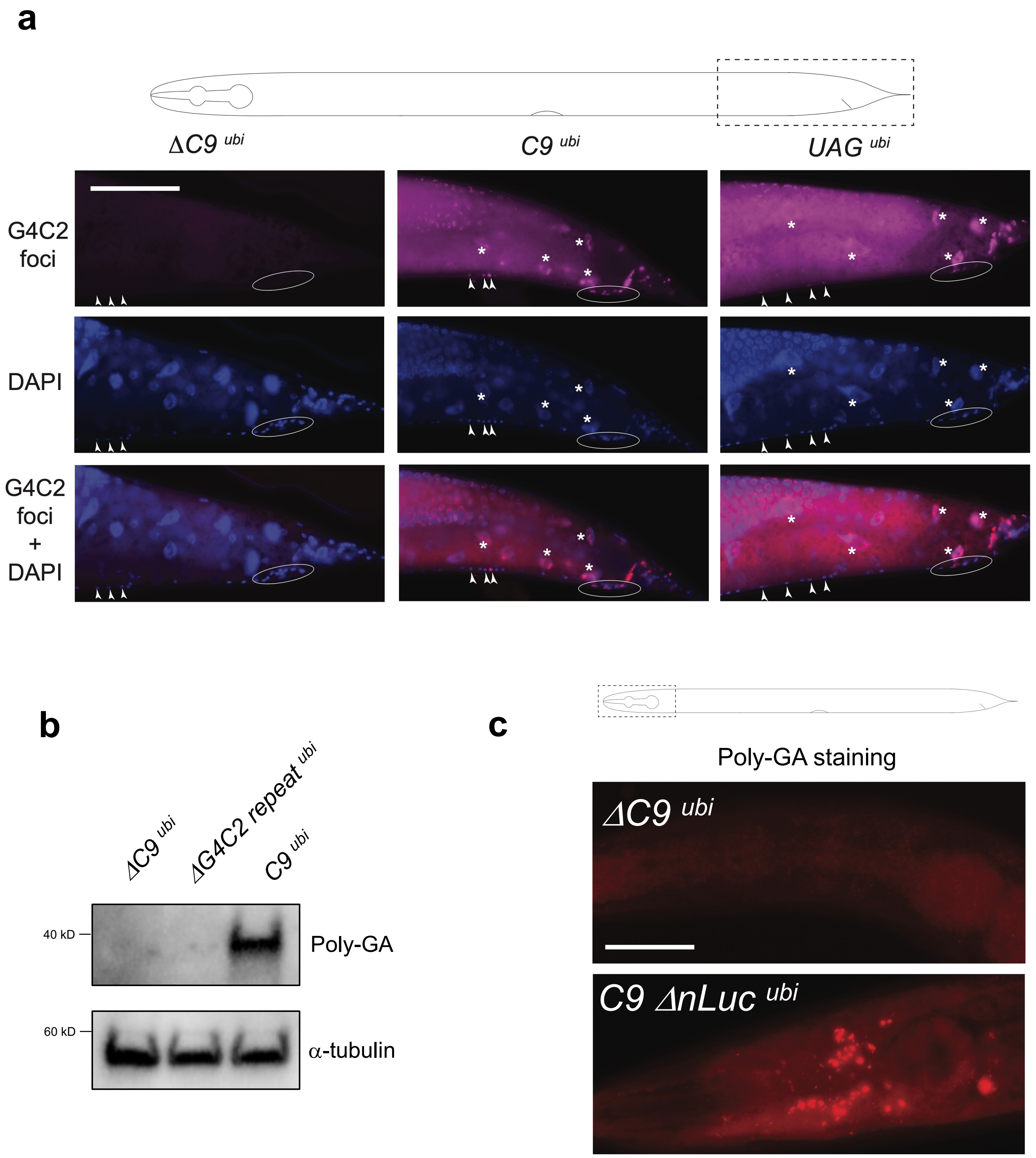

Supplementary Figure 3: G4C2 RNA foci are detected in neuronal and non-neuronal cells of C9^{ubi} and UAG^{ubi} animals. (a) Representative fluorescent images of the tail of adult (day 1) *ΔC9*^{ubi} (*kasIs9*), *C9*^{ubi} (*kasIs7*) and *UAG*^{ubi} (*kasIs10*) animals. N = 25. G4C2 RNA foci are visualized with a Quasar 670 probe. Nuclei are demarcated with DAPI (blue). G4C2 RNA foci are detected in motor neurons of the ventral nerve cord (arrowheads) and the preanal ganglion (circled) both in *C9*^{ubi} and *UAG*^{ubi} animals. RNA foci are also detected in other cells, including intestinal cells (asterisks). (b) Lysates were processed for Western blotting from *ΔC9*^{ubi}, *ΔG4C2 repeat*^{ubi}, and *C9*^{ubi} worms and immunostained with poly-GA and α-tubulin antibodies. Experiments were repeated 3 times. (c) *ΔC9*^{ubi} *(KasEx154)* and *C9 ΔnLuc*^{ubi} *(KasEx237)* animals (day 1) were immunostained for poly-GA (red). Representative images of the head region taken at 40x magnification. N = 20. Anterior to the left. Scale bar = 25 μm.

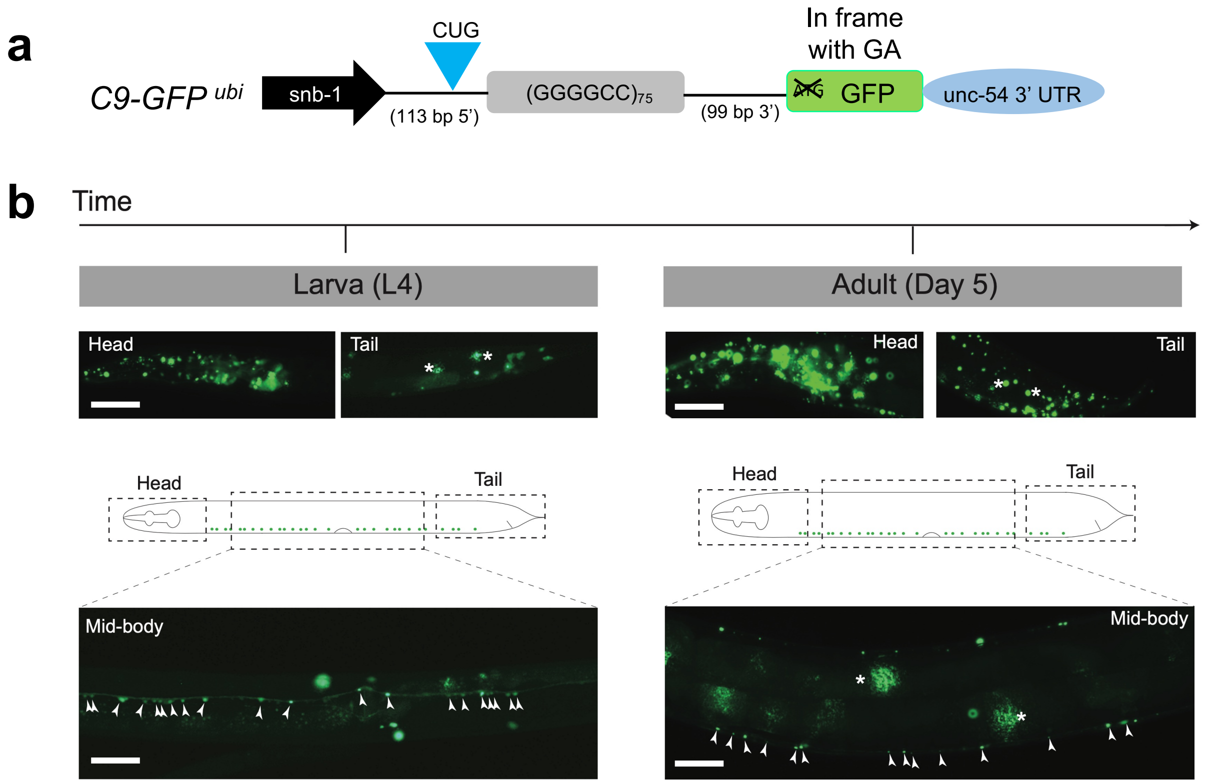

Supplementary figure 4. Poly-GA accumulation over time and effect of UAG mutation on DPR production (a) Schematic of construct used to generate the *C9-GFP* ^{ubi} animals. This is the same construct as *C9* ^{ubi} animals in Figure 1, but green fluorescent protein (GFP), not nLuc, is in poly-GA frame. UTR: untranslated region. (b) Representative images of head, tail and mid-body regions of *C9-GFP* ^{ubi} animals at larval (L4) and adult (day 5) stages. Aggregated GFP puncta (proxy for poly-GA expression) observed in head and tail neurons, as well as ventral nerve cord motor neurons (arrowheads). Aggregated GFP puncta were also present in non-neuronal cells, such as muscle and intestine (asterisks). GFP accumulation is more evident in adult (day 5) than L4 *C9-GFP*^{ubi} animals. Two independent transgenic lines *kasEx247 [Psnb-1::intron-75G4C2 repeats-intron-GFPnovo2]* and *kasEx252 [Psnb-1::intron-75G4C2 repeats-intron-GFPnovo2]* were analyzed. N = 17 per line per time point. Scale bar = 25 µm.

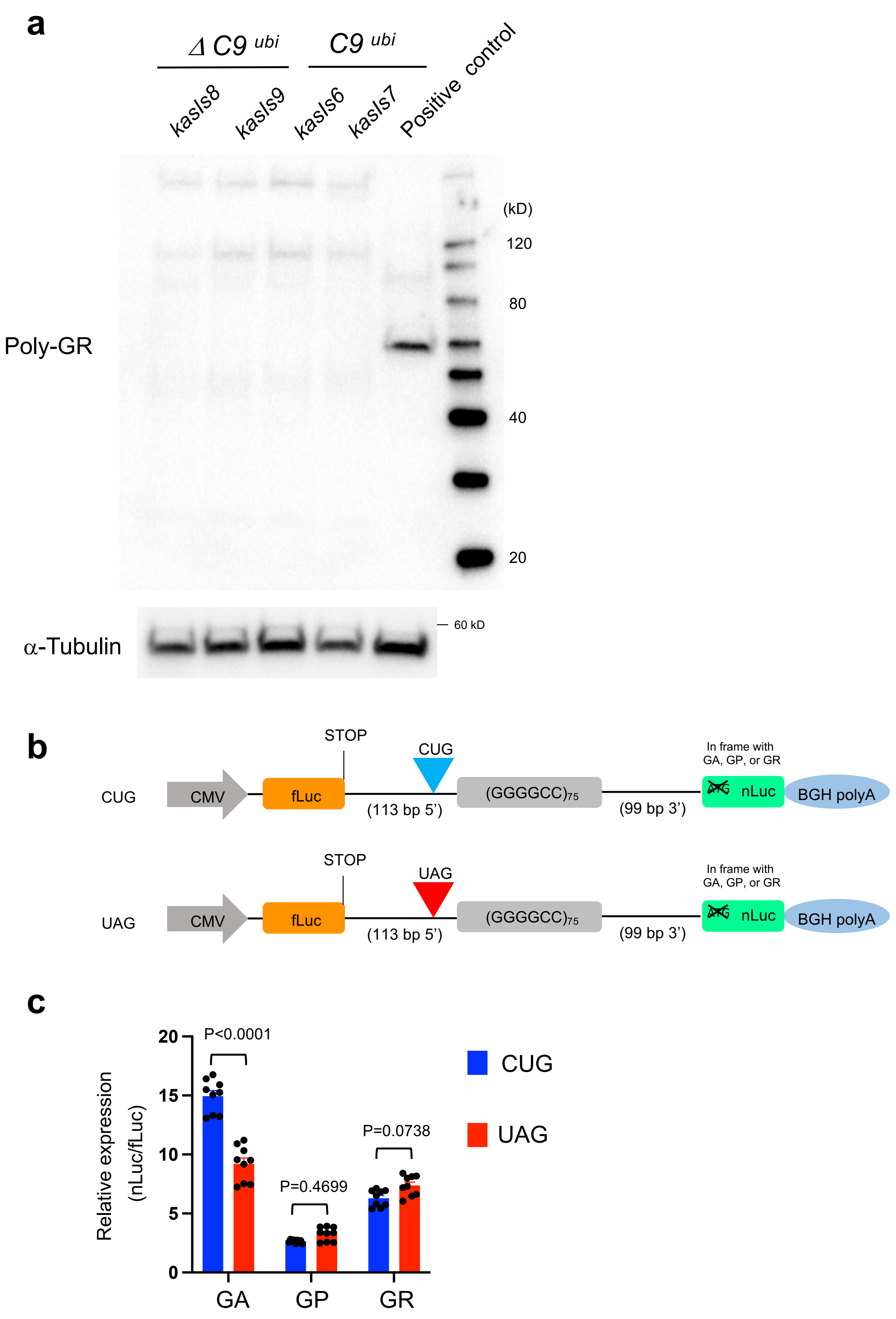

Supplementary figure 5. Western blots to detect poly-GR in *C. elegans* and effect of CUG mutation on DPRs in HEK293 cells. (a) The lysates from $\Delta C9^{ubi}$ *(KasIs8, KasIs9)* and $C9^{ubi}$ *(KasIs6, KasIs7)* worms were processed for Western blotting and immunostained with poly-GR and α-tubulin antibodies. Positive control: HEK293 cells transfected with GR^{AUG}(G4C2$_{75}$-nLuc) plasmid, in which an AUG start codon was inserted before the G4C2 repeats and in the reading frame of poly-GR. (b-c) Bicistronic constructs including CUG or UAG were transfected into HEK 293 cells for 48h. (b) Schematic diagram showing bicistronic constructs. CMV: cytomegalovirus; fLuc: firefly luciferase; nLuc: nanoluciferase; BGH: bovine growth hormone. (c) The cell lysates were processed for luciferase assay. The levels of luciferase activity were assessed by dual luciferase assays. The experiments were repeated 3 times. mean ± s.e.m. Two-way ANOVA with Šídák's multiple comparison test was performed.

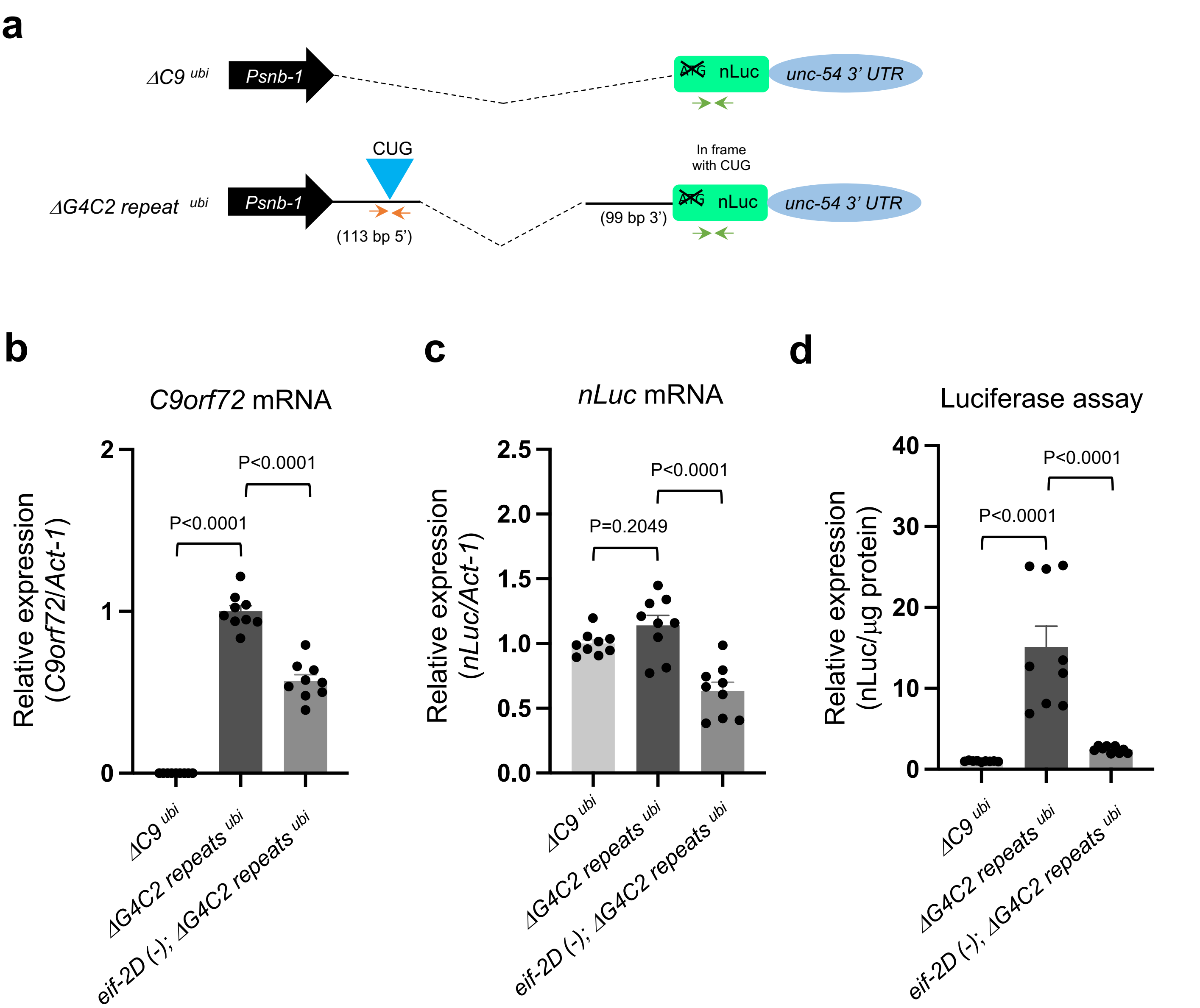

Supplementary figure 6. Analyses of nLuc mRNA and protein in *eif-2D* mutant animals carrying the *ΔG4C2 repeat* ^{ubi} transgene. (a) Schematic diagram showing *ΔC9* ^{ubi} *(KasEx154)* and *ΔG4C2 repeat* ^{ubi} *(KasEx240)* constructs. The orange and green arrows show primer location to detect *C9orf72* and nLuc mRNAs, respectively. nLuc: nanoluciferase; UTR: untranslated region. (b-c) Act-1, *C9orf72* (intronic) and nLuc mRNAs were assessed by RT-PCR. (d) A luciferase assay was performed on worm lysates. The *eif-2D (gk904876)* allele was used. Experiments were repeated 3 times. mean ± s.e.m. One-way ANOVA with Dunnett's multiple comparisons test was performed.

a

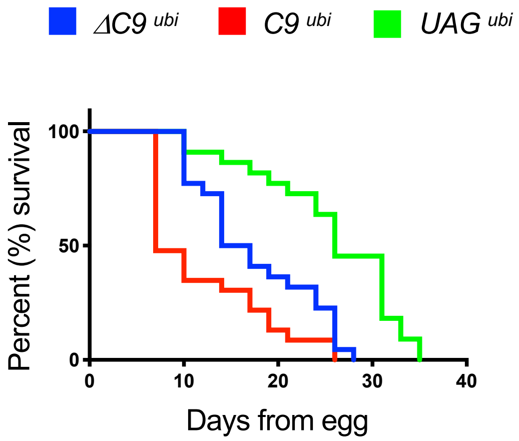

Replicate 1

	ΔC9 ubi	*C9 ubi*	*UAG ubi*
Animals included	22	23	22
Censored animals	3	2	3
Median Lifespan (Days)	15.5	7	26

b

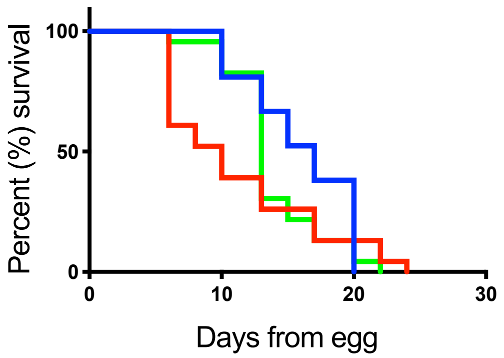

Replicate 2

	ΔC9 ubi	*C9 ubi*	*UAG ubi*
Animals included	21	23	23
Censored animals	4	2	2
Median Lifespan (Days)	17	10	13

c

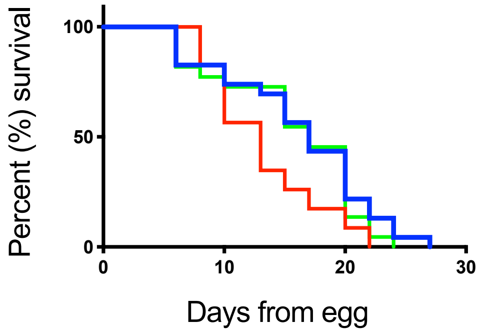

Replicate 3

	ΔC9 ubi	*C9 ubi*	*UAG ubi*
Animals included	23	23	22
Censored animals	2	2	3
Median Lifespan (Days)	17	13	17

d

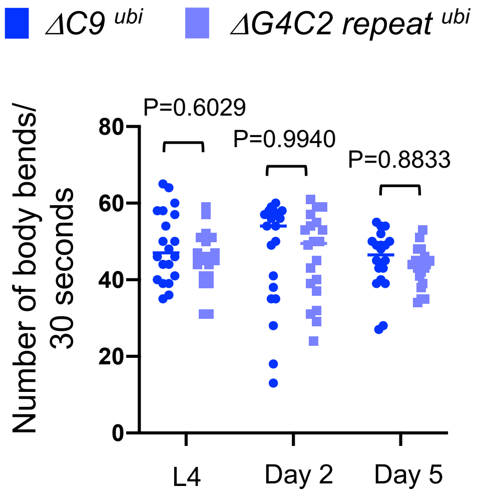

Summary of 3 replicates

	ΔC9 ubi	*C9 ubi*	*UAG ubi*
Animals included	66	68	67
Censored animals	9	7	8
Median Lifespan (Days)	17	10	17

ΔC9 ubi vs C9 ubi; P<0.0001

C9 ubi vs UAG ubi; P<0.0001

Supplementary figure 7. Replicates of the lifespan assay shown in Figure 2a. (a-c) The survival graph and data tables for 3 replicates of the survival assay shown in Fig. 2a. The strains used in each replicate are as follows. Replicate 1: ΔC9 *ubi* (*KasEx154*), *C9 ubi (kasEx261), UAG ubi* (*KasEx264*); Replicate 2: *(ΔC9 ubi (KasEx259)*, C9 *ubi (KasEx262)*, *UAG ubi (KasEx155),* Replicate 3: *ΔC9 ubi (KasEx260), C9 ubi ; KasEx153, UAG ubi (KasEx263*). (d) Rate of body flexion measure on *ΔC9 ubi* and *ΔG4C2 repeat ubi* animals at the indicated time points. N=20. Two-way ANOVA with Šídák's multiple comparison test was performed.

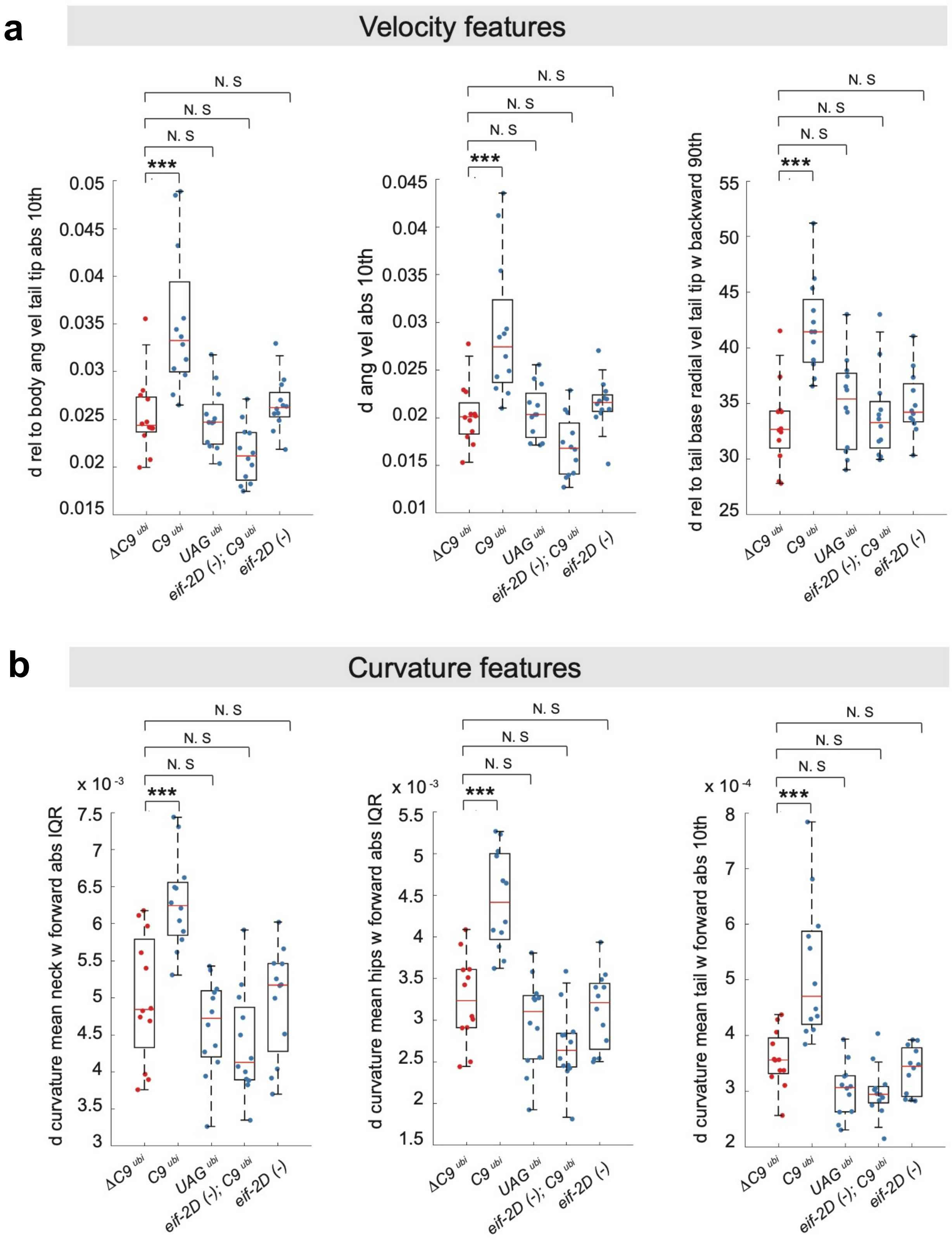

Supplementary figure 8. Genetic removal of *eif-2D/eIF2D* ameliorates the locomotor defects of *C9* ^{ubi} animals. Examples of locomotion features significantly affected in *C9* ^{ubi} animals. Genetic removal of *eif-2D* ameliorated the locomotion defects of *C9* ^{ubi} animals [*eif-2D (-); C9* ^{ubi}]. Tracking analysis was performed at Day 2 adult animals (N = 12). Unpaired two-sided t-tests were performed. The threshold for significance was set using the Benjamini-Yekutieli procedure with a false discovery rate of 0.05. The p-value threshold for this analysis (FDR 5%) is 0.0014 (see Methods). The boxes show the median and the 25th and 75th percentiles. The whisker length is set to either 1.5 times the interquartile range or the distance to the maximum and minimum sample points, whichever is greater. (a) Three locomotion features related to animal velocity. *d rel to body ang vel tail tip abs 10th*: 10th percentile of the derivative of the absolute value of the angular velocity of the tip of the tail relative to the centroid of the mid-body points. *d ang vel abs 10*th: 10th percentile of the derivative of the absolute value of the angular velocity of the worm. *d rel to tail base radial vel tail tip w backward 90*th*:* 90th percentile of the derivative of the radial velocity of the tip of the tail relative to the centroid of the tail base points, while the worm is moving backwards. (b) Three locomotion features related to body curvature while the worm is moving forward. *d curvature mean neck w forward abs IQR*: interquartile range of the derivative of the absolute value of the mean curvature of the neck. *d curvature mean hips w forward abs IQR*: interquartile range of the derivative of the absolute value of the mean curvature of the hips. *d curvature mean tail w forward abs 10*th: 10th percentile of the derivative of the absolute value of the mean curvature of the tail. Lines: *ΔC9* ^{ubi} *(KasEx154), C9* ^{ubi} *(KasEx153), UAG* ^{ubi} *(KasEx155).*

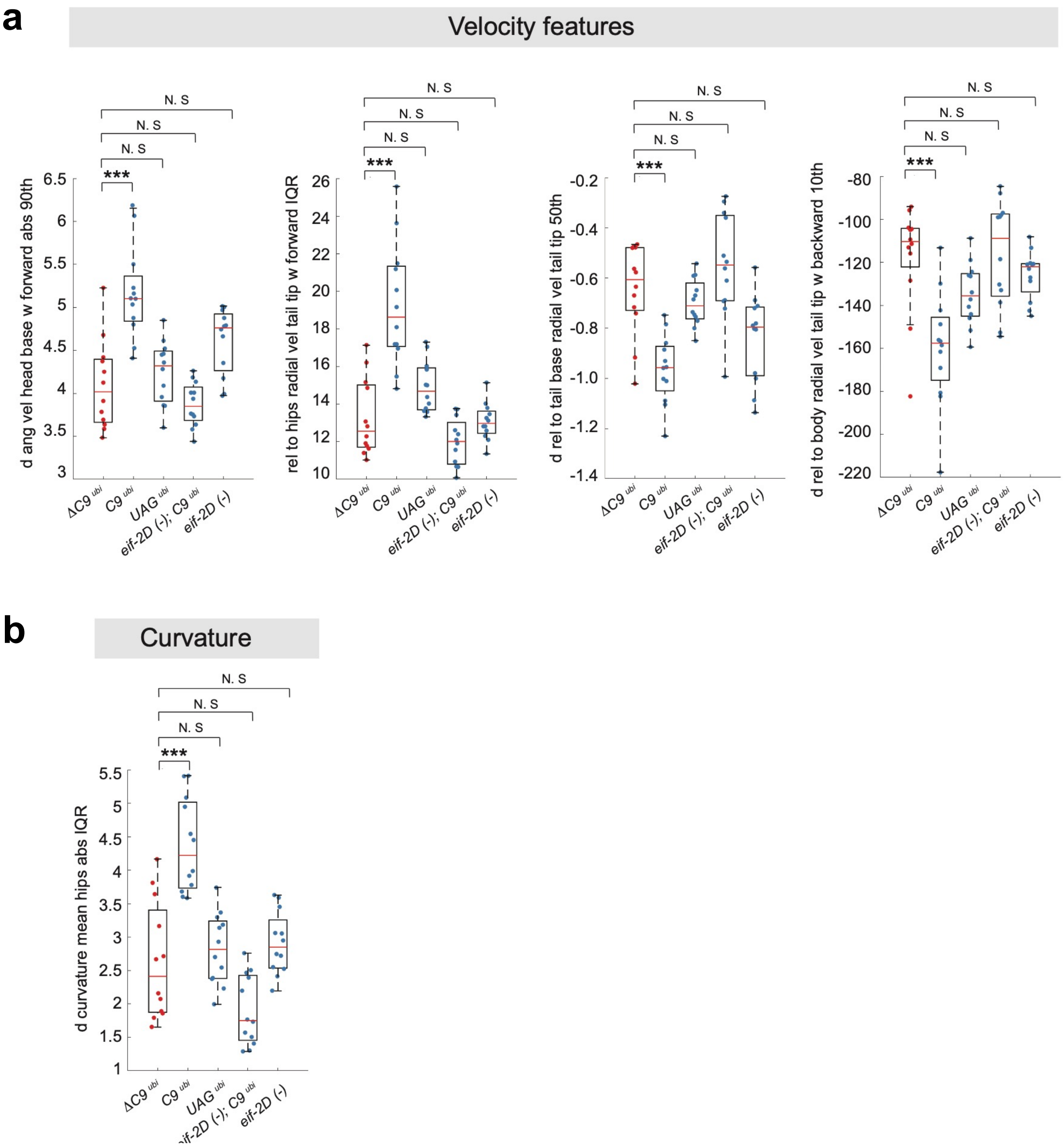

Supplementary figure 9. Defects in velocity and body curvature are ameliorated in *C9 ubi* animals upon genetic removal of *eif-2D/eIF2D*.

Examples of seven additional locomotion features significantly affected in *C9 ubi* animals. Genetic removal of *eif-2D* ameliorated the locomotion defects of *C9 ubi* animals (*eif-2D (-); C9 ubi)*. Tracking analysis was performed at Day 2 adult animals (N = 12). Unpaired two-sided t-tests were performed. The threshold for significance was set using the Benjamini-Yekutieli procedure with a false discovery rate of 0.05. The p-value threshold for this analysis (FDR 5%) is 0.0014 (see Methods). The boxes show the median and the 25th and 75th percentiles. The whisker length is set to either 1.5 times the interquartile range or the distance to the maximum and minimum sample points, whichever is greater. (a) Features related to animal velocity. *d ang vel head base w forward abs 90th:* 90th percentile of the derivative of the absolute value of the angular velocity of the base of the head, while the worm is moving forwards. *rel to hips radial vel tail tip w forward IQR:* interquartile range of the radial velocity of the tip of the head relative to the centroid of the hips points, while the worm is moving forwards. *d rel to tail base radial vel tail tip 50th:* 50th percentile of the derivative of the radial velocity of the tip of the tail relative to the centroid of the tail base points. *d rel to body radial vel tail tip w backward 10th:* 10th percentile of the derivative of radial velocity of the tip of the tail relative to the centroid of the midbody points, while the worm is moving backwards. (b) A feature related to body curvature. *d curvature mean hips abs IQR:* interquartile range of the derivative of the absolute value of the mean curvature of the hips. Lines: *ΔC9 ubi (KasEx154), C9 ubi (KasEx153), UAG ubi (KasEx155).*

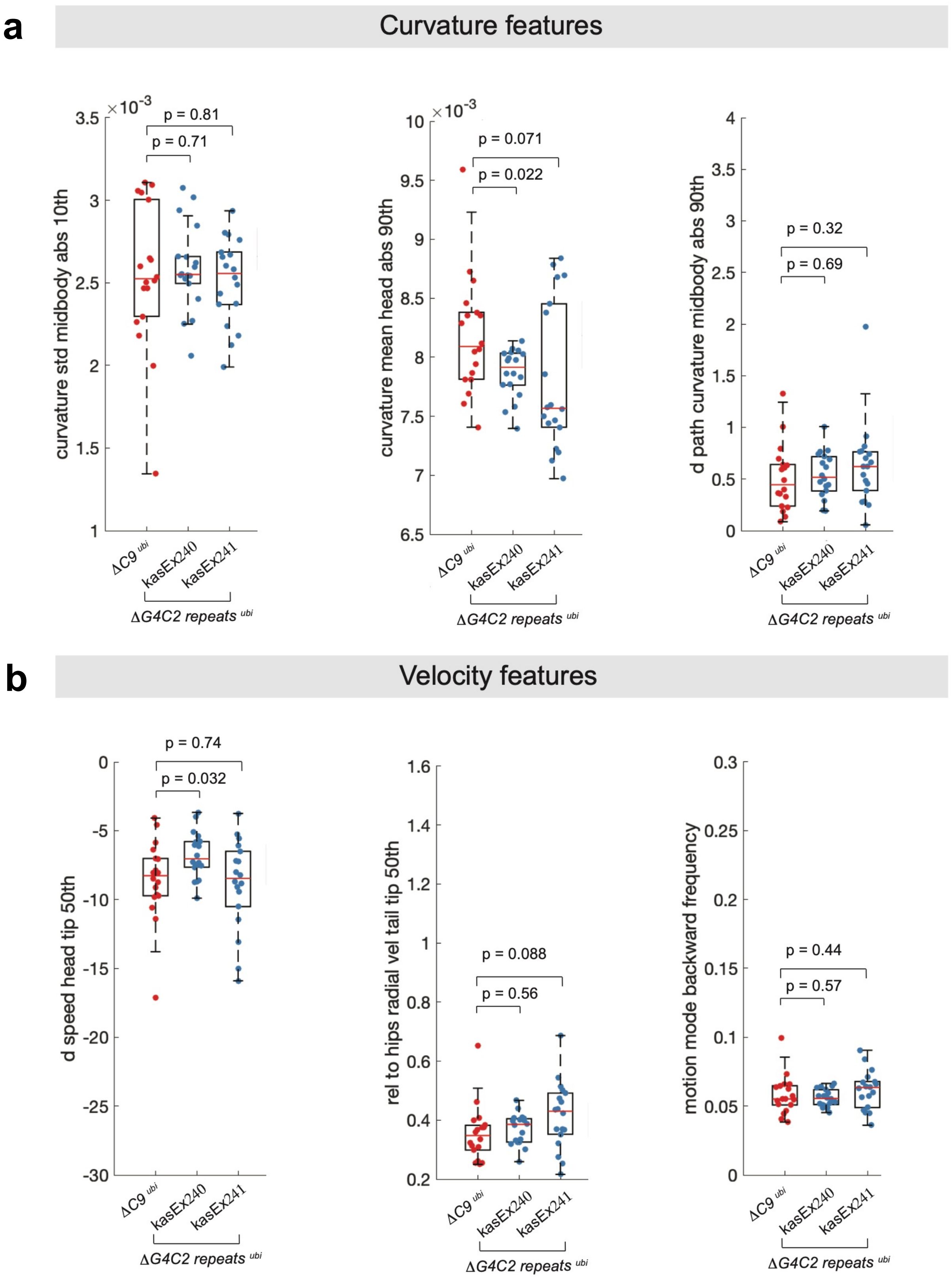

Supplementary figure 10. Curvature and velocity features appear normal in ΔC9^{ubi} and ΔG4C2 repeats^{ubi} animals. Examples of six locomotion features not affected in ΔC9^{ubi} and *ΔG4C2 repeats*^{ubi} *animals*. Tracking analysis was performed at Day 2 adult animals. Lines *kasEx154 (ΔC9*^{ubi}*)* and *kasEx240-1 (ΔG4C2 repeats*^{ubi}*)* were used. N = 15 animals per line. The boxes show the median and the 25th and 75th percentiles. The whisker length is set to either 1.5 times the interquartile range or the distance to the maximum and minimum sample points, whichever is greater. Unpaired two-sided t-tests were performed. The threshold for significance was set using the Benjamini-Yekutieli procedure with a false discovery rate of 0.05. The p-value threshold for this analysis (FDR 5%) is 0.0014 (see Methods). (a) Features related to body curvature. *curvature_std_midbody_abs_10*th*:* 10th percentile of the absolute value of the standard deviation of the curvature of the midbody. *curvature_mean_head_abs_90*th: 90th percentile of the absolute value of the mean curvature of the head. *d_path_curvature_midbody_abs_90*th: 90th percentile of the derivative of the absolute value of the curvature of the path of the midbody. (b) Features related to velocity and frequency of movement. *d_speed_head_tip_50*th: 50th percentile of the derivative of the speed of the tip of the head. *rel_to_hips_radial_vel_tail_tip_50*th*:* 50th percentile of the radial velocity of the tip of the head relative to the centroid of the hips points. *motion_mode_backward_frequency*: frequency that a worm is moving backwards.

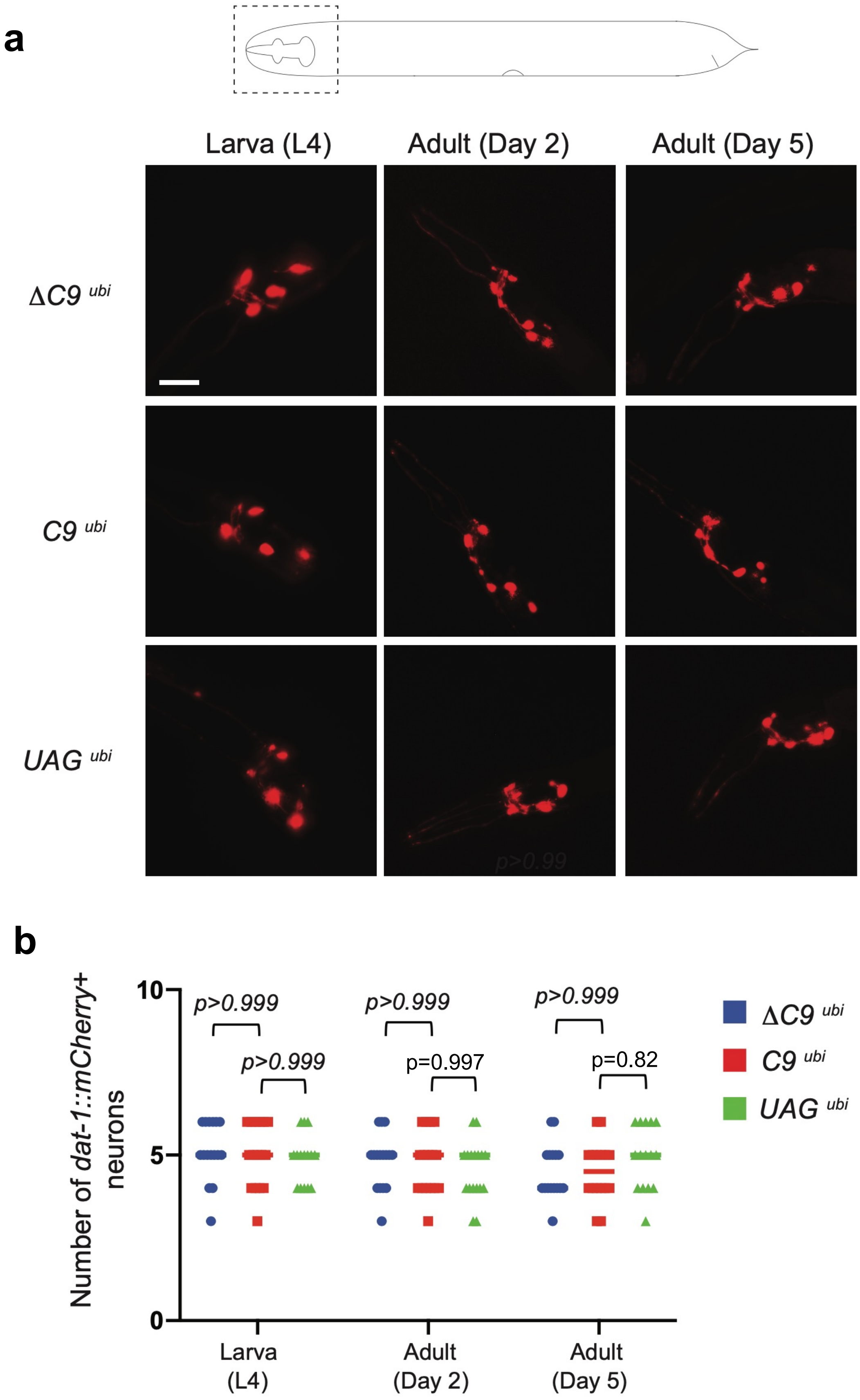

Supplementary figure 11. The number of dopaminergic neurons is unaffected in C9 *ubi* animals. (a) Representative images of the head of *ΔC9 ubi* (*kasIs9*), *C9 ubi* (*kasIs7*) and *UAG ubi* (*kasIs10*) animals carrying the dopaminergic marker *otIs181[dat-1::mCherry]*. (b) Quantification was performed at three different stages (L4, day 2, day 5). N= 16. Two-way ANOVA with Šídák's multiple comparison test was performed. Scale bar = 25 µm.

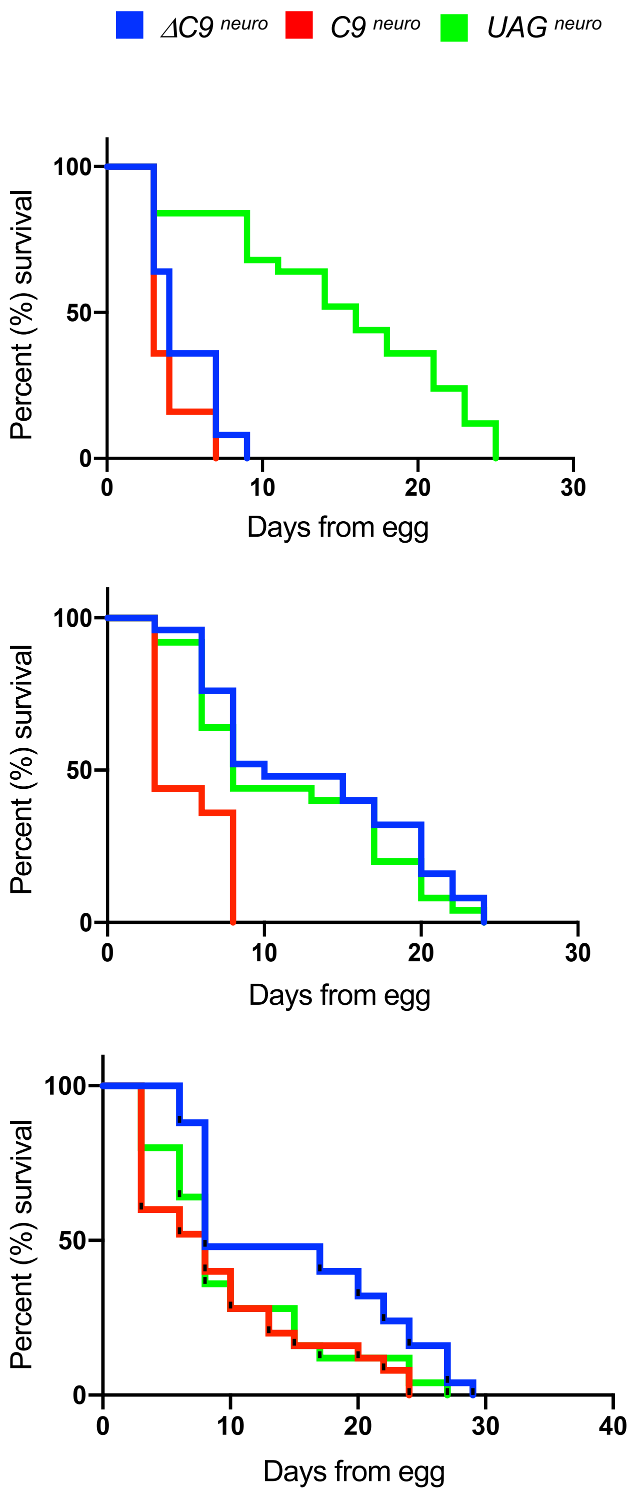

Replicate 1

	ΔC9 *neuro*	C9 *neuro*	UAG *neuro*
Number of animals included	25	25	25
Number of censored animals	3	3	3
Median lifespan (Days)	4	3	16

Replicate 2

	ΔC9 *neuro*	C9 *neuro*	UAG *neuro*
Number of animals included	25	25	25
Number of censored animals	3	3	3
Median lifespan (Days)	10	3	8

Replicate 3

	ΔC9 *neuro*	C9 *neuro*	UAG *neuro*
Number of animals included	25	25	25
Number of censored animals	3	3	3
Median lifespan (Days)	8	8	8

Summary of 3 replicates

	ΔC9 *neuro*	C9 *neuro*	UAG *neuro*
Number of animals included	75	75	75
Number of censored animals	9	9	9
Median lifespan (Days)	8	3	9

ΔC9 neuro vs C9 neuro; P<0.0001
C9 neuro vs UAG neuro; P<0.0001

Supplementary figure 12. Replicates of the lifespan assay shown in Figure 3f. The survival graph and data tables for 3 replicates shown in Fig. 3f. The strains used in each replicate were as follows: Replicate 1-3 (ΔC9 *neuro*: *KasEx159*, C9 *neuro*: *KasEx157*, UAG *neuro*: *KasEx160*). Mantel-Cox log rank test was performed.

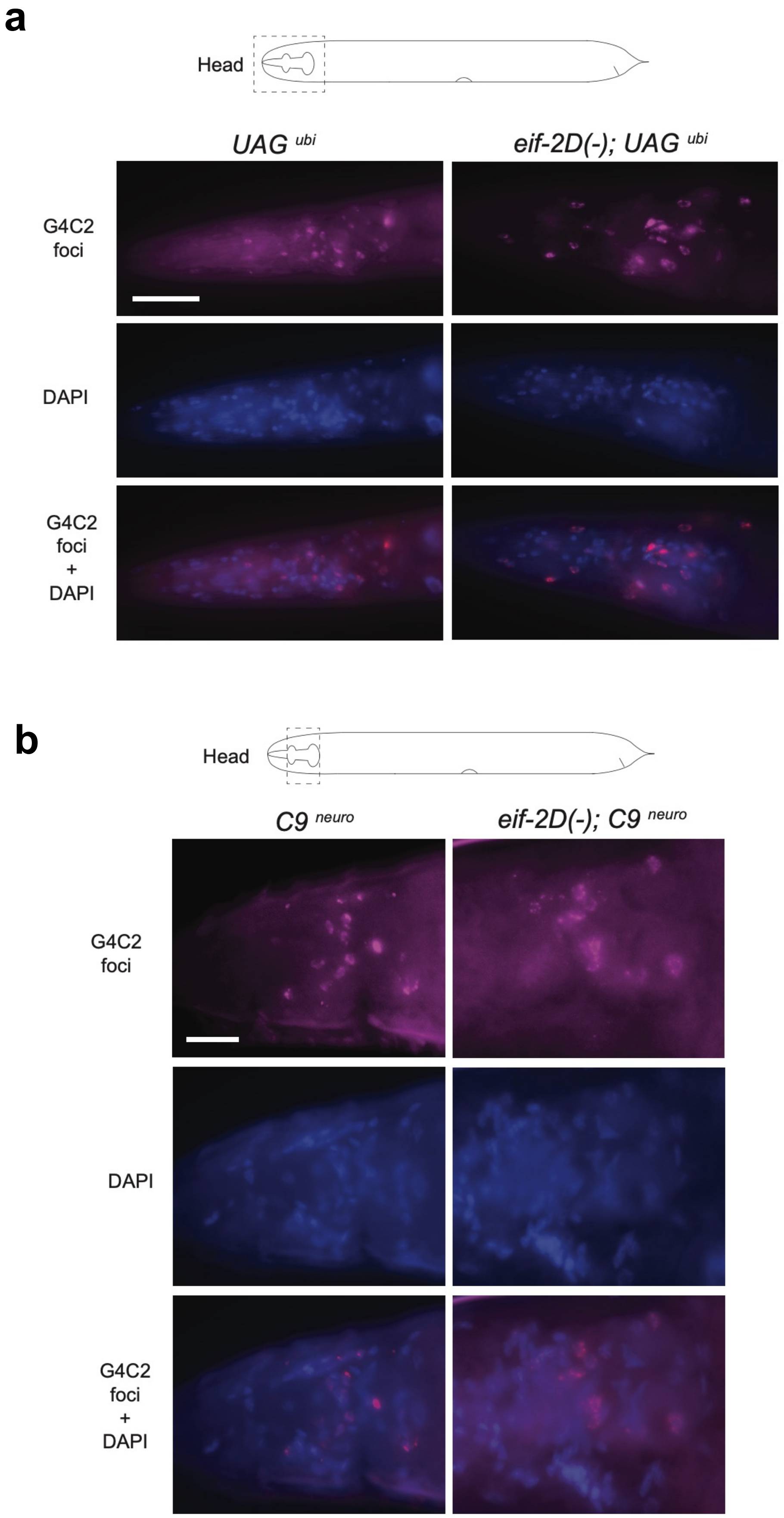

Supplementary figure 13. G4C2 RNA foci are detected in cells of *eif-2D* mutant animals.

(a) Representative fluorescent images of the head of adult (day 1) *UAG* ^{ubi} (*kasEx155*) and *eif-2D (gk904876); UAG* ^{ubi} (*kasEx155*) animals. N = 20. G4C2 RNA foci are visualized with a Quasar 670 probe. Nuclei are demarcated with DAPI (blue). Scale bar = 25μm. (b) Representative fluorescent images of the head of adult (day 1) *C9* ^{neuro} (*kasEx157*) and *eif-2D (gk904876); C9* ^{neuro} (*kasEx157*) animals. N = 20. G4C2 RNA foci are visualized with a Quasar 670 probe. Nuclei are demarcated with DAPI (blue). Scale bar = 10 μm.

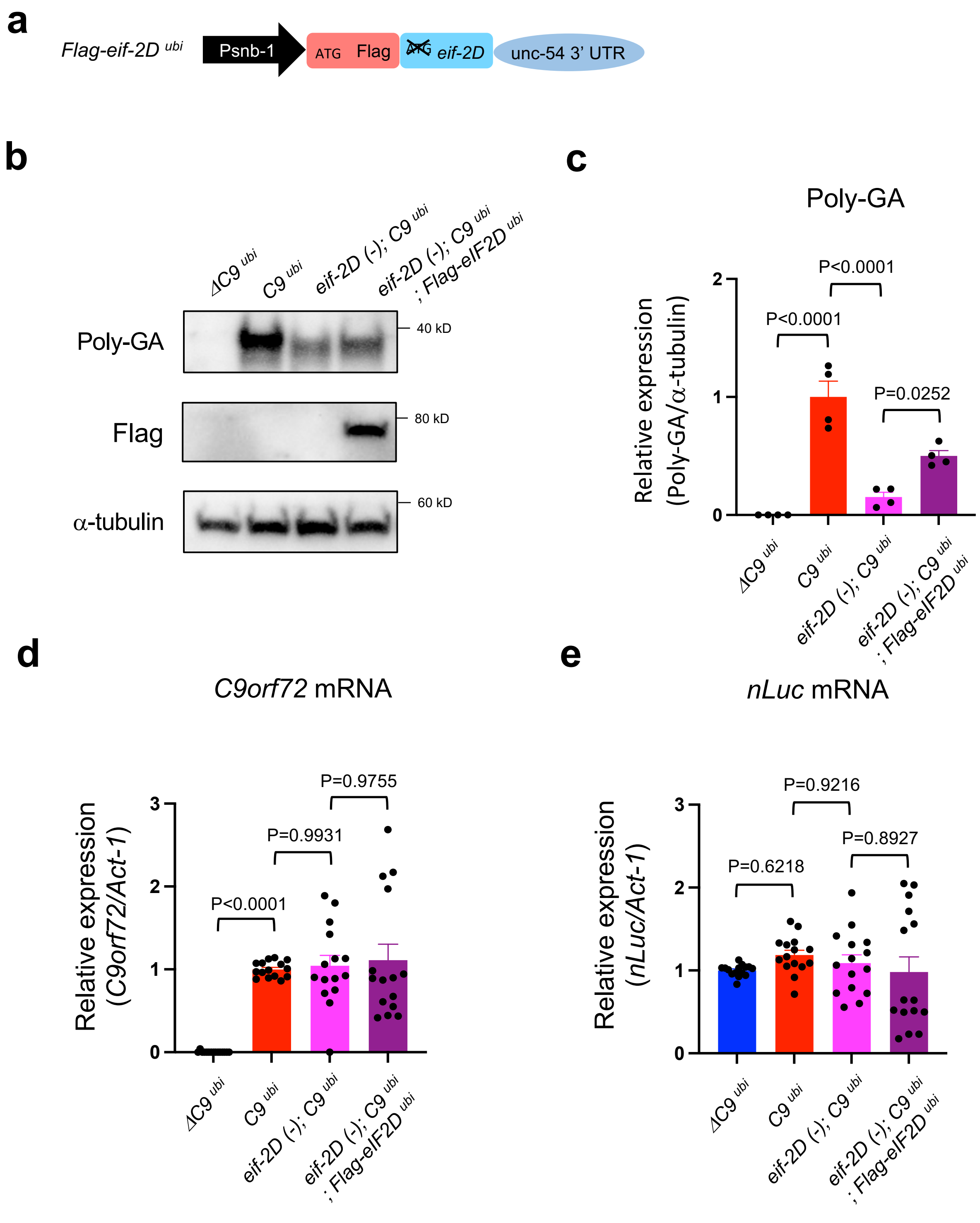

Supplementary figure 14. Expression of *eif-2D* partially restores poly-GA expression in *eif-2D (gk904876)* mutant worms. (a) Schematic diagram showing *Flag-eif-2D* construct used to generate *KasEx248* transgenic animals. (b) The worm lysates were processed for Western blotting. Representative Western blot immunostained with poly-GA and α-tubulin antibodies. (c) Quantification of poly-GA on Western blots. The experiments were repeated 4 times. (d) The 75 G4C2 repeat RNA (with *C9orf72* intronic RNA) and Act-1 mRNA were assessed by RT-PCR. (e) The *nLuc* and Act-1 mRNAs were assessed by RT-PCR. The experiments were repeated 5 times. Mean ± s.e.m. One-way ANOVA with Tukey's multiple comparisons test was performed. The C9 ^{ubi} (*KasEx153*) line was used. nLuc: nanoluciferase; UTR: untranslated region.

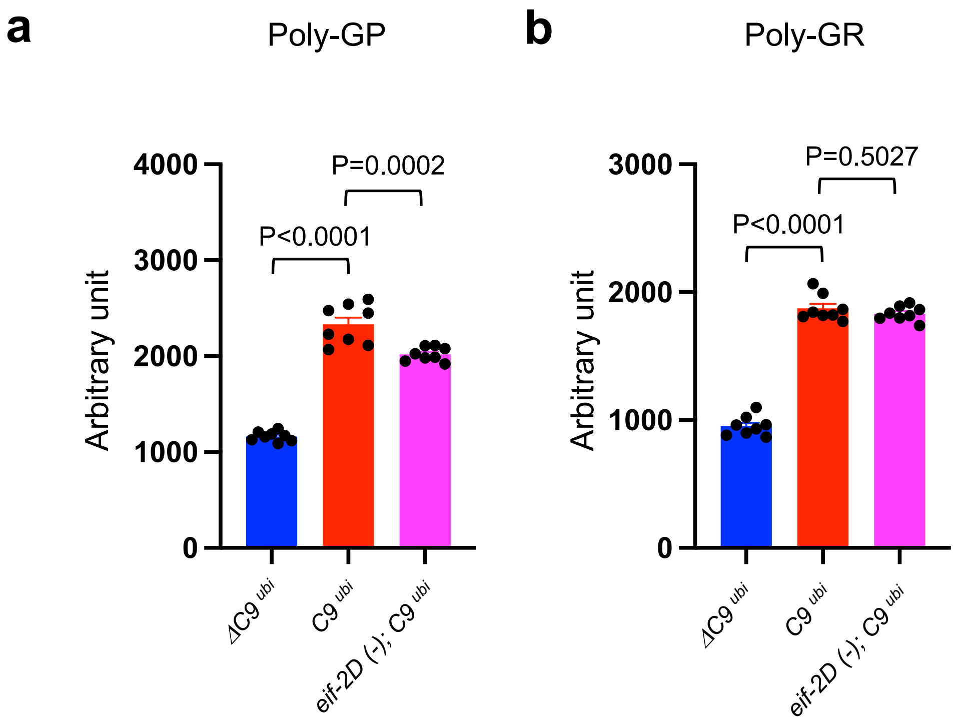

Supplementary figure 15. The level of poly-GP is reduced in *eif-2D (gk904876)* mutant worms. The lysates from *ΔC9* ^{ubi}, *C9* ^{ubi} *(KasEx153)* and *eif-2D (-)*; C9 ^{ubi} worms were processed for (a) poly-GP and (b) poly-GR ELISA. The experiments were repeated 4 times, mean ± s.e.m. One-way ANOVA with Dunnett's multiple comparisons test was performed.

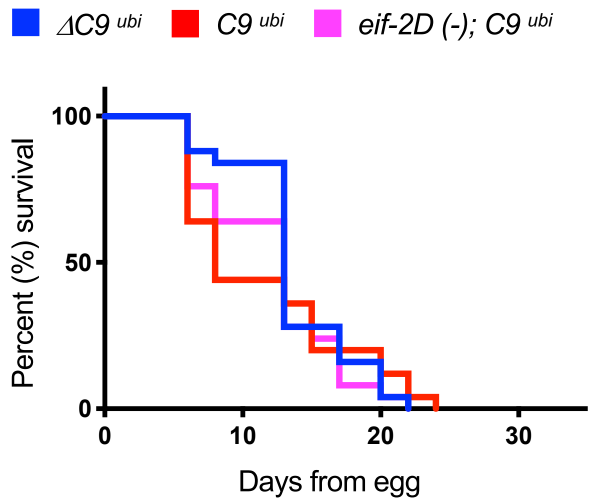

Replicate 1

	$\Delta C9^{ubi}$	$C9^{ubi}$	*eif-2D (-);* $C9^{ubi}$
Number of animals included	25	25	25
Number of censored animals	2	2	2
Median lifespan (Days)	13	8	13

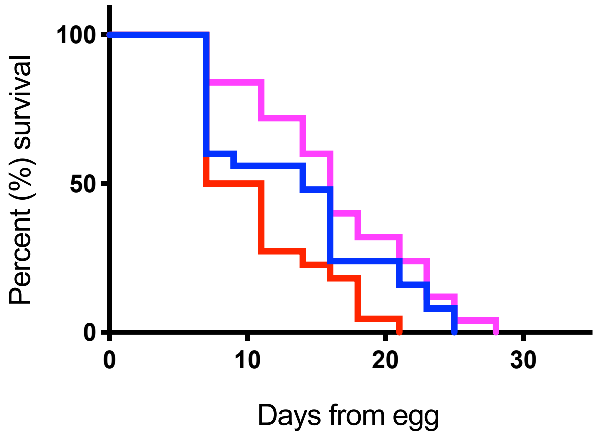

Replicate 2

	$\Delta C9^{ubi}$	$C9^{ubi}$	*eif-2D (-);* $C9^{ubi}$
Number of animals included	25	22	25
Number of censored animals	2	5	2
Median lifespan (Days)	13	8	13

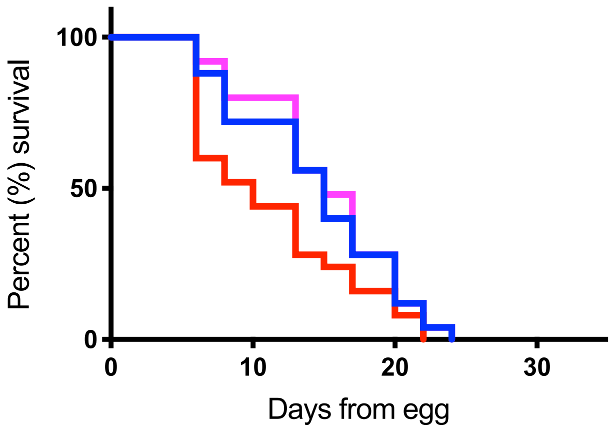

Replicate 3

	$\Delta C9^{ubi}$	$C9^{ubi}$	*eif-2D (-);* $C9^{ubi}$
Number of animals included	25	25	25
Number of censored animals	2	2	2
Median lifespan (Days)	15	10	15

$\Delta C9^{ubi}$ *vs* $C9^{ubi}$*;* $P<0.0079$

$C9^{ubi}$ *vs eif-2D (-);* $C9^{ubi}$*;* $P<0.0017$

Summary of 3 replicates

	$\Delta C9^{ubi}$	$C9^{ubi}$	*eif-2D (-);* $C9^{ubi}$
Number of animals included	75	72	75
Number of censored animals	6	9	6
Median lifespan (Days)	13	8	14

Supplementary figure 16. Replicates of the lifespan assay shown in Figure 4e. The survival graph and data tables for 3 replicates shown in Fig. 4e. The strains used in each replicate were as follows. Replicates 1-3: $\Delta C9^{ubi}$ *(KasEx154),* $C9^{ubi}$*(kasEx262).* Mantel-Cox log rank test was performed.

Summary of 2 replicates

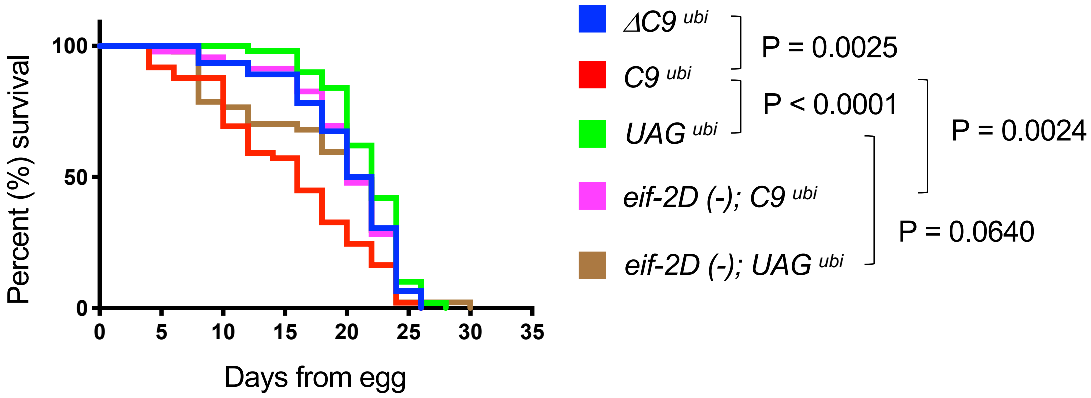

	ΔC9 ubi	*C9 ubi*	*UAG ubi*	*eif-2D (-); C9 ubi*	*eif-2D (-); UAG ubi*
Number of animals included	46	49	50	46	47
Number of censored animals	4	1	0	4	3
Median lifespan (Days)	21	16	22	20	20

Replicate 1

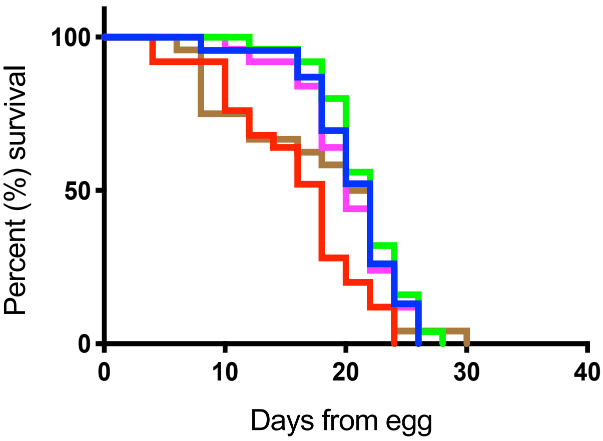

	ΔC9 ubi	*C9 ubi*	*UAG ubi*	*eif-2D (-); C9 ubi*	*eif-2D (-); UAG ubi*
Number of animals included	23	25	25	25	24
Number of censored animals	2	0	0	0	1
Median lifespan (Days)	22	18	22	20	21

Replicate 2

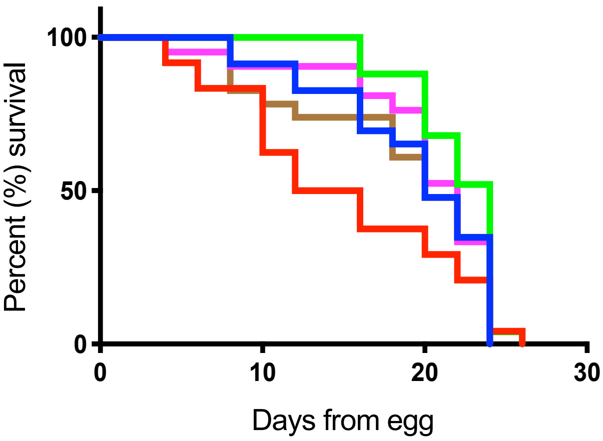

	ΔC9 ubi	*C9 ubi*	*UAG ubi*	*eif-2D (-); C9 ubi*	*eif-2D (-); UAG ubi*
Number of animals included	23	24	25	21	23
Number of censored animals	2	1	0	4	2
Median lifespan (Days)	20	14	24	22	20

Supplementary figure 17. *C9 ubi* animals display a significantly shorter lifespan in assays without FUDR. The lifespan assays and data tables for *ΔC9 ubi (KasEx154), C9 ubi (KasEx153), UAG ubi (KasEx155), eif-2D (-); C9 ubi* and *eif-2D (-); UAG ubi* animals are shown. The experiments were repeated twice. Mantel-Cox log rank test was performed. Details for the individual experiments are shown in lower panels.

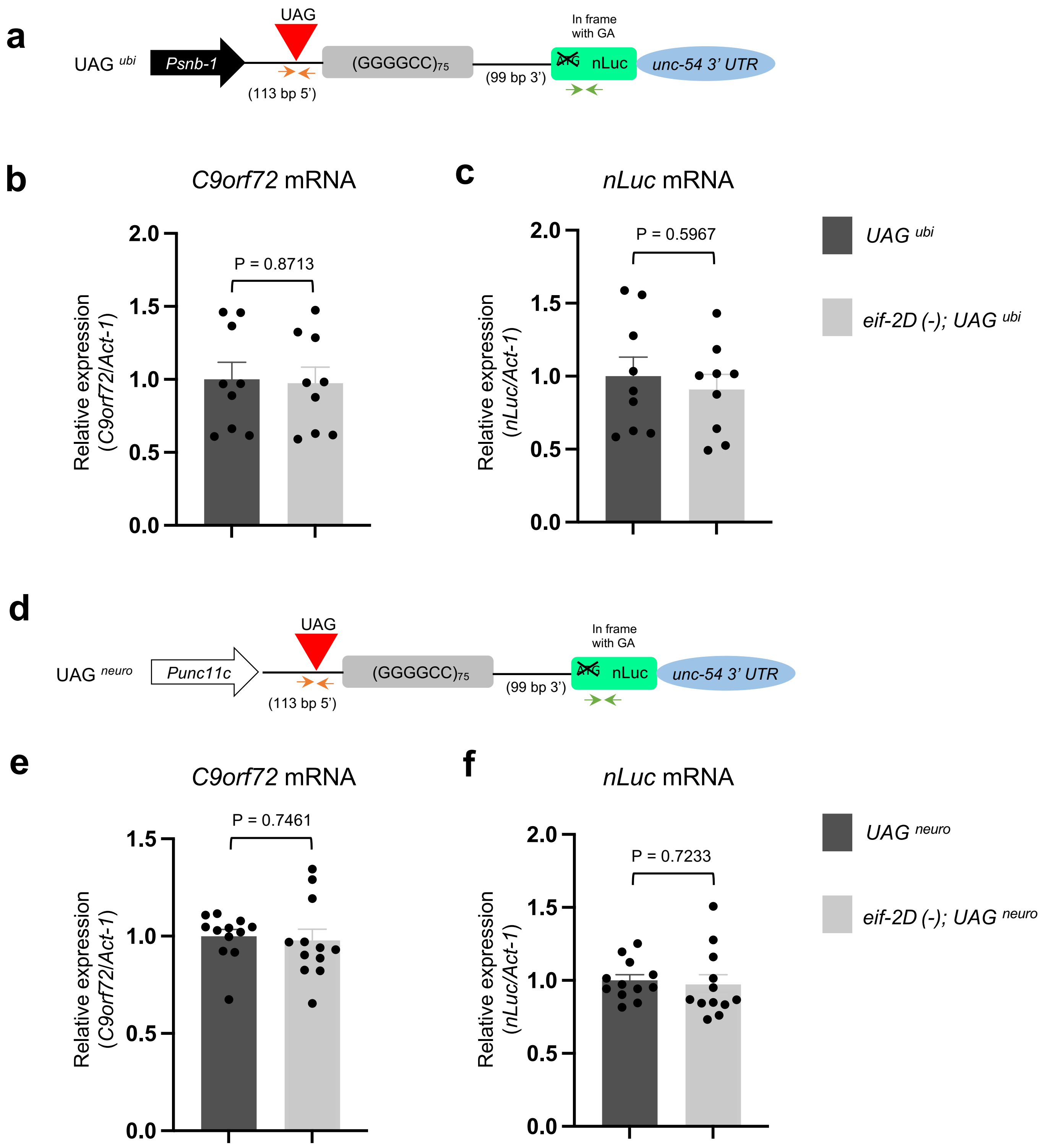

Supplementary figure 18. Knockout of *eif-2D* does not affect the levels of *C9orf72* intronic RNA and *nLuc* mRNAs in both UAG ^{ubi} and UAG ^{neuro} animals. (a, d) Schematic diagram showing UAG constructs. The orange and green arrows show the location of primers designed to detect *C9orf72* and nLuc mRNAs, respectively. nLuc: nanoluciferase; UTR: untranslated region. The *C9orf72* intronic RNA (b, e) and *nLuc* mRNAs (c, f) were assessed by RT-PCR and normalized to Act-1 mRNA. The experiments in b-c were repeated 3 times, and the experiments in e-f were repeated 4 times. Mean ± s.e.m. Two-tailed unpaired t-test was performed. Lines used: *UAG ^{ubi} (KasEx155), UAG ^{neuro} (KasEx161).*

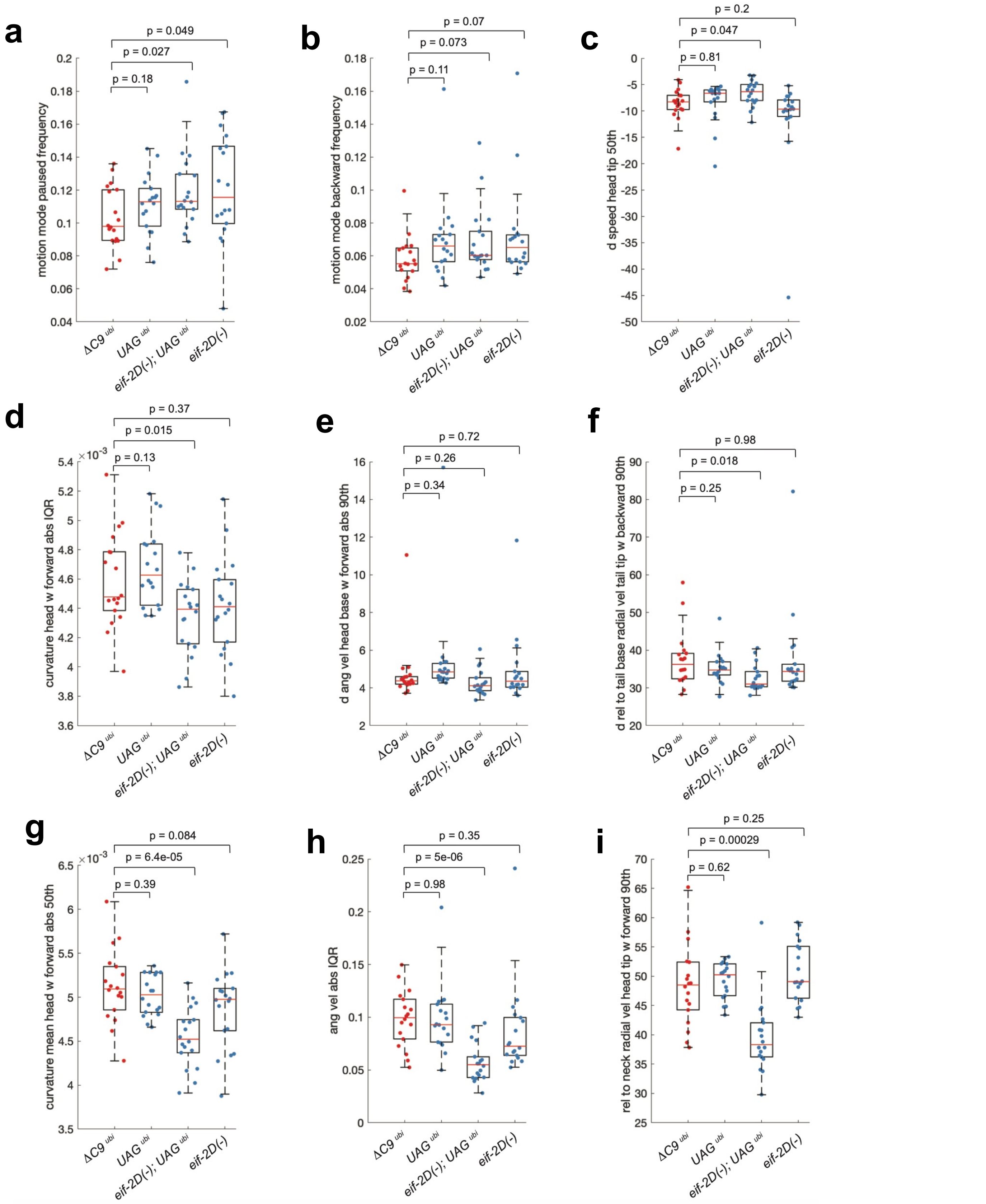

Supplementary figure 19. Locomotion features appear normal in *ΔC9 ubi*, *UAG ubi*, *eif-2D(gk904876); UAG ubi* and *eif-2D (gk904876)* animals. Examples of nine locomotion features (a-i) assessed in *ΔC9 ubi*, *UAG ubi*, *eif-2D(-); UAG ubi*, and *eif-2D(-)* animals. Tracking analysis was performed at Day 2 adult animals. Transgenic lines *kasEx154 (ΔC9 ubi)* and *kasEx155 (UAG ubi)* were used. N = 15 animals per line. Unpaired two-sided t-tests were performed. The threshold for significance was set using the Benjamini-Yekutieli procedure with a false discovery rate of 0.05. The p-value threshold (after correcting for multiple comparisons) is 0.00096. The boxes show the median and the 25th and 75th percentiles. The whisker length is set to either 1.5 times the interquartile range or the distance to the maximum and minimum sample points, whichever is greater. The following locomotion features are shown from top left to bottom right: *motion_mode_paused_frequency*: frequency that a worm is paused (not moving); *motion_mode_backward_frequency:* frequency that a worm is moving backwards; *d_speed_head_tip_50th*: 50th percentile of the derivative of the speed of the tip of the head; *curvature_head_w_forward_abs_IQR*: interquartile range of the absolute value of the curvature of the head, while the worm is moving forwards; *d_ang_vel_head_base_w_forward_abs_90th*: 90th percentile of the derivative of the absolute value of the angular velocity of the base of the head, while the worm is moving forwards; *d_rel_to_tail_base_radial_vel_tail_tip_w_backward_90th*: 90th percentile of the derivative of the radial velocity of the tip of the tail relative to the centroid of the tail base points, while the worm is moving backwards; *curvature_mean_head_w_forward_abs_50th*: 50th percentile of the absolute value of the mean curvature of the head, while the worm is moving forwards; *ang_vel_abs_IQR*: interquartile range of the absolute value of the angular velocity of the worm; *rel_to_neck_radial_vel_head_tip_w_forward_90th*: 90th percentile of the radial velocity of the tip of the head relative to the centroid of the neck points, while the worm is moving forwards. Lines used: *ΔC9 ubi (KasEx154), C9 ubi (KasEx153).*

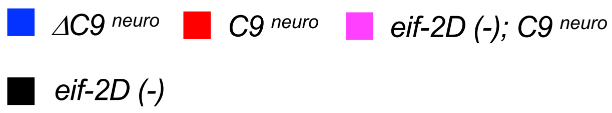

Replicate 1

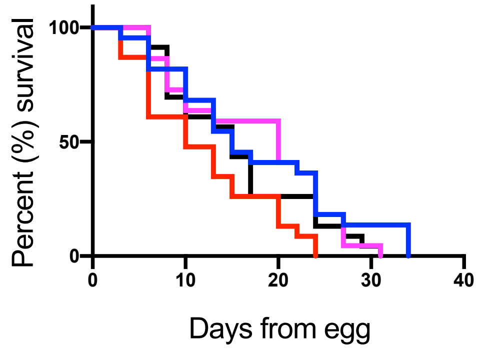

	ΔC9^{neuro}	*C9^{neuro}*	*eif-2D (-); C9^{neuro}*	*eif-2D (-)*
Number of animals included	22	23	22	23
Number of censored animals	3	2	3	2
Median lifespan (Days)	15	10	20	15

Replicate 2

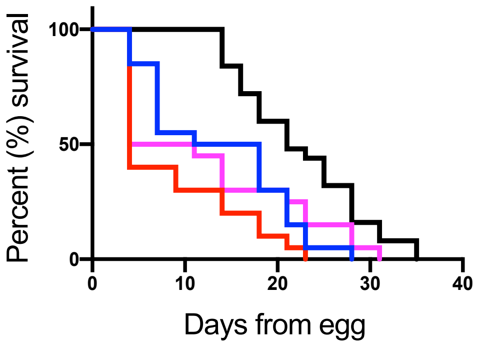

	ΔC9^{neuro}	*C9^{neuro}*	*eif-2D (-); C9^{neuro}*	*eif-2D (-)*
Number of animals included	20	20	20	25
Number of censored animals	5	5	5	0
Median lifespan (Days)	14.5	4	7.5	21

Replicate 3

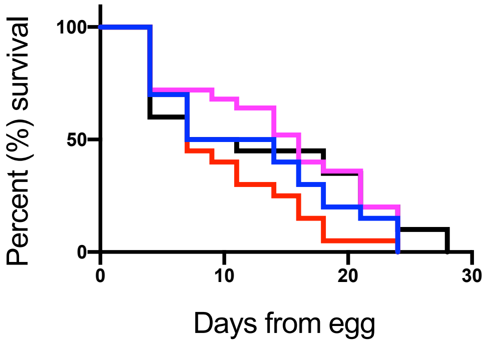

	ΔC9^{neuro}	*C9^{neuro}*	*eif-2D (-); C9^{neuro}*	*eif-2D (-)*
Number of animals included	20	20	25	20
Number of censored animals	5	5	0	5
Median lifespan (Days)	10.5	7	16	9

Summary of 3 replicates

	ΔC9^{neuro}	*C9^{neuro}*	*eif-2D (-); C9^{neuro}*	*eif-2D (-)*
Number of animals included	62	63	67	68
Number of censored animals	13	12	8	7
Median lifespan (Days)	14	7	14	17

ΔC9^{neuro} vs C9^{neuro}; P=0.0009

C9^{neuro} vs eif-2D (-); C9^{neuro}; P=0.0001

ΔC9^{neuro} vs eif-2D (-); P=0.0678

Supplementary figure 20. Replicates of the lifespan assay shown in Figure 4j. The survival graph and data tables for 3 replicates shown in Fig. 4j. The strains used in each replicate were as follows. Replicate 1-3: *ΔC9^{neuro} (KasEx159), C9^{neuro} (KasEx157).* Mantel-Cox log rank test was performed.

Summary of 3 replicates

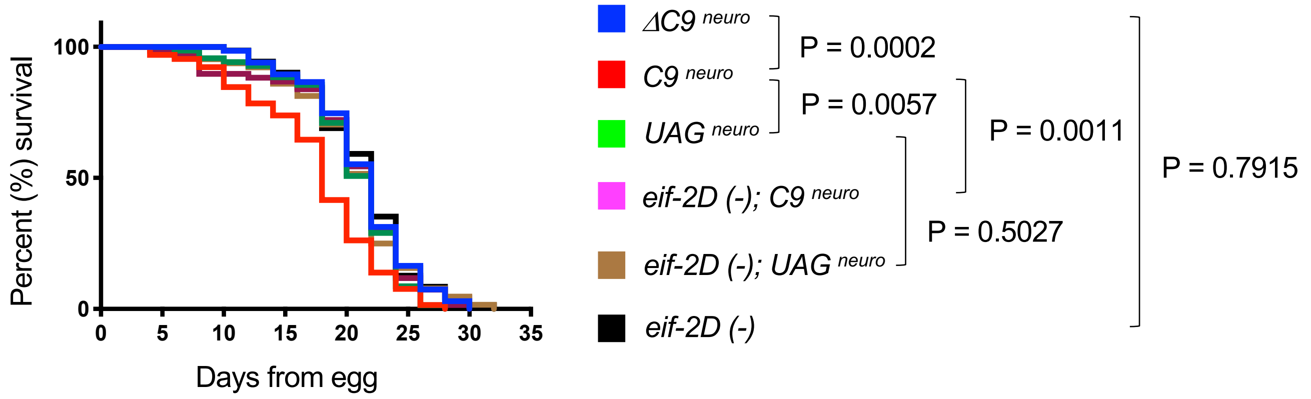

	ΔC9 neuro	*C9 neuro*	*UAG neuro*	*eif-2D (-); C9 neuro*	*eif-2D (-); UAG neuro*	*eif-2D (-)*
Number of animals included	67	65	69	68	64	71
Number of censored animals	8	10	6	7	11	4
Median lifespan (Days)	22	18	22	22	22	22

Replicate 1

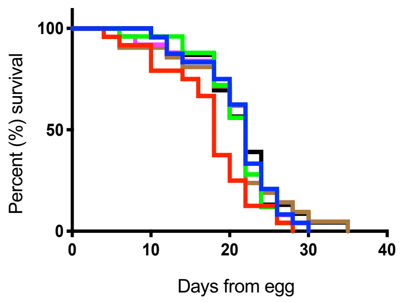

	ΔC9 neuroi	*C9 neuro*	*UAG neuro*	*eif-2D (-); C9 neuro*	*eif-2D (-); UAG neuro*	*eif-2D (-)*
Number of animals included	24	24	25	25	21	23
Number of censored animals	1	1	0	0	4	2
Median lifespan (Days)	22	18	22	22	22	22

Replicate 2

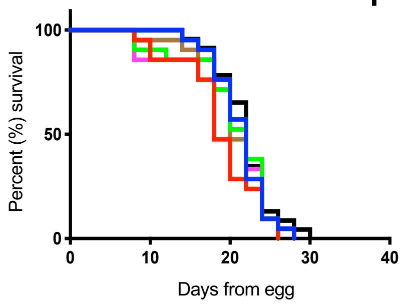

	ΔC9 neuro	*C9 neuro*	*UAG neuro*	*eif-2D (-); C9 neuro*	*eif-2D (-); UAG neuro*	*eif-2D (-)*
Number of animals included	21	21	21	21	21	23
Number of censored animals	4	4	4	4	4	2
Median lifespan (Days)	22	18	22	22	20	22

Replicate 3

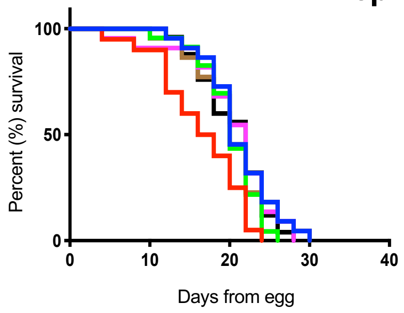

	ΔC9 neuro	*C9 neuro*	*UAG neuro*	*eif-2D (-); C9 neuro*	*eif-2D (-); UAG neuro*	*eif-2D (-)*
Number of animals included	22	20	23	22	22	25
Number of censored animals	4	4	4	4	4	2
Median lifespan (Days)	20	17	20	22	20	22

Supplementary figure 21. *C9 neuro* animals display a significantly shorter lifespan in assays without FUDR. The lifespan assays and data tables for *ΔC9 neuro (KasEx159), C9 neuro (KasEx157), UAG neuro (KasEx161), eif-2D (gk904876); C9 neuro, eif-2D (gk904876); UAG neuro* and *eif-2D (gk904876)* animals are shown. The experiments were repeated 3 times. Details for the individual experiments are shown in lower panels. Mantel-Cox log rank test was performed.

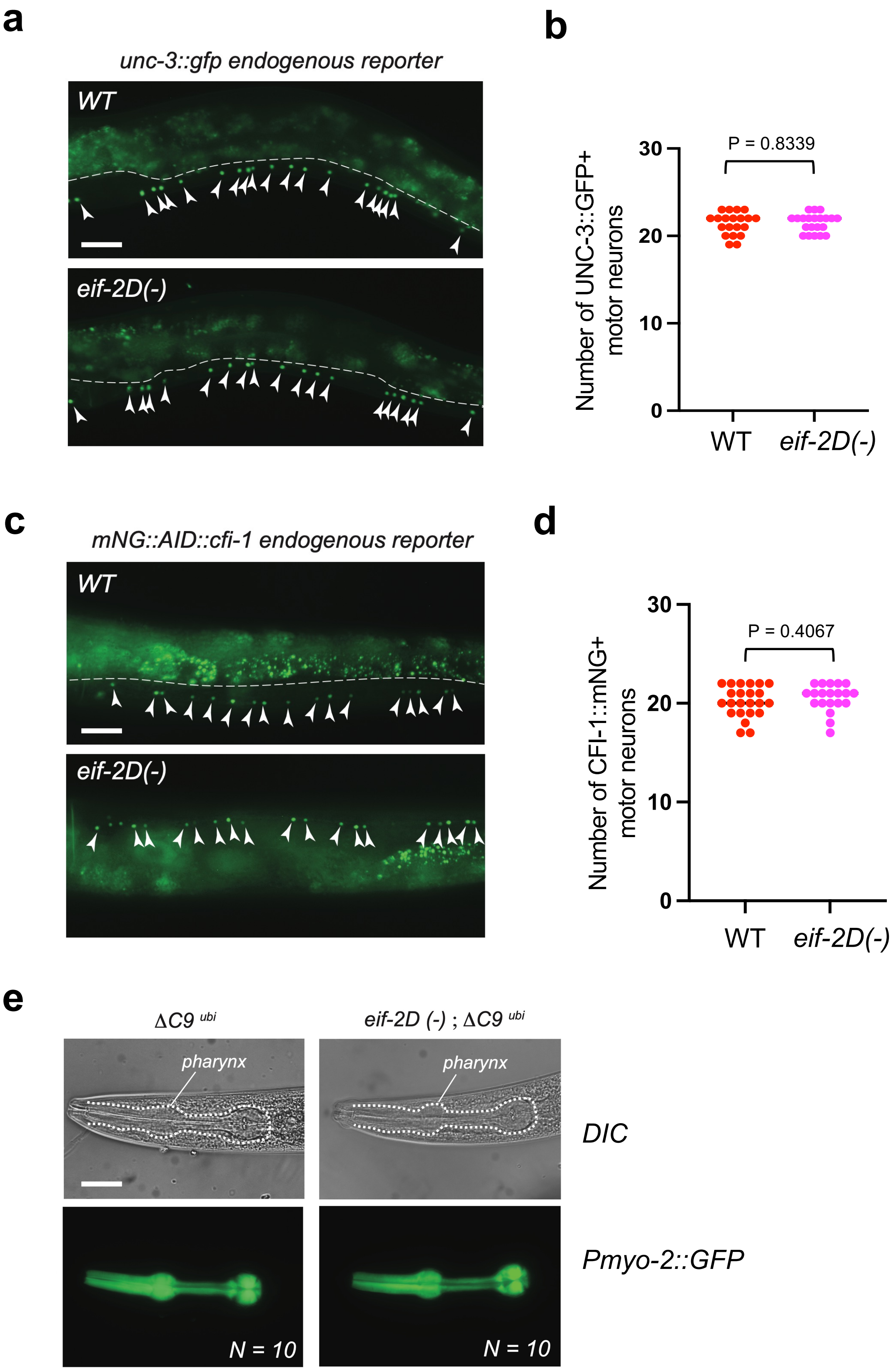

Supplementary figure 22. Genetic removal of *eif-2D* does not affect the endogenous expression of two transcription factors and an AUG-initiated transgene. (a) Representative images of wild-type (WT) and *eif-2D (gk904876)* animals (L4 stage) carrying an endogenous *gfp* reporter allele for the *unc-3* transcription factor *(ot839 [unc-3::gfp])*. Scale bar = 10 µm. WT: N = 20, *eif-2D(-)*: N = 21. (b) Quantification of the number of motor neurons expressing UNC-3::GFP in the posterior half of the ventral nerve cord. (c) Representative images of wild-type (WT) and *eif-2D (gk904876)* animals (day 1 adult) carrying an endogenous *mNeonGreen (mNG)* reporter allele for the *cfi-1* transcription factor *(kas16 [mNG::AID::cfi-1])*. Scale bar = 10 µm. (d) Quantification of the number of motor neurons expressing CFI-1::mNG in the posterior half of the ventral nerve cord. The *unc-3 (ot839 [unc-3::gfp]*) and *cfi-1 (kas16 [mNG::AID::cfi-1])* alleles generate protein fusions. Only the motor neurons located between the vulva and the preanal ganglion were counted. WT: N = 23, *eif-2D(-)*: N = 20. Two-tailed unpaired t-test was performed. Nuclear expression in motor neurons is indicated with arrowheads. Anterior is to the left. White dotted line delineates the boundary of the gut, which is autofluorescent in the green channel. (e) The ΔC9 *ubi* transgene carries an AUG-initiated *gfp* marker (*myo-2::gfp*) and is expressed in the pharynx. This expression is not affected in *eif-2D (gk904876)* animals. Representative images of larval stage 4 (L4) worms are shown. Scale bar = 25 µm. Differential interference contrast (DIC) images of the worm head are shown on top. Dashed white line demarcates the pharynx. Fluorescent images are shown below in the GFP channel. N = 10.

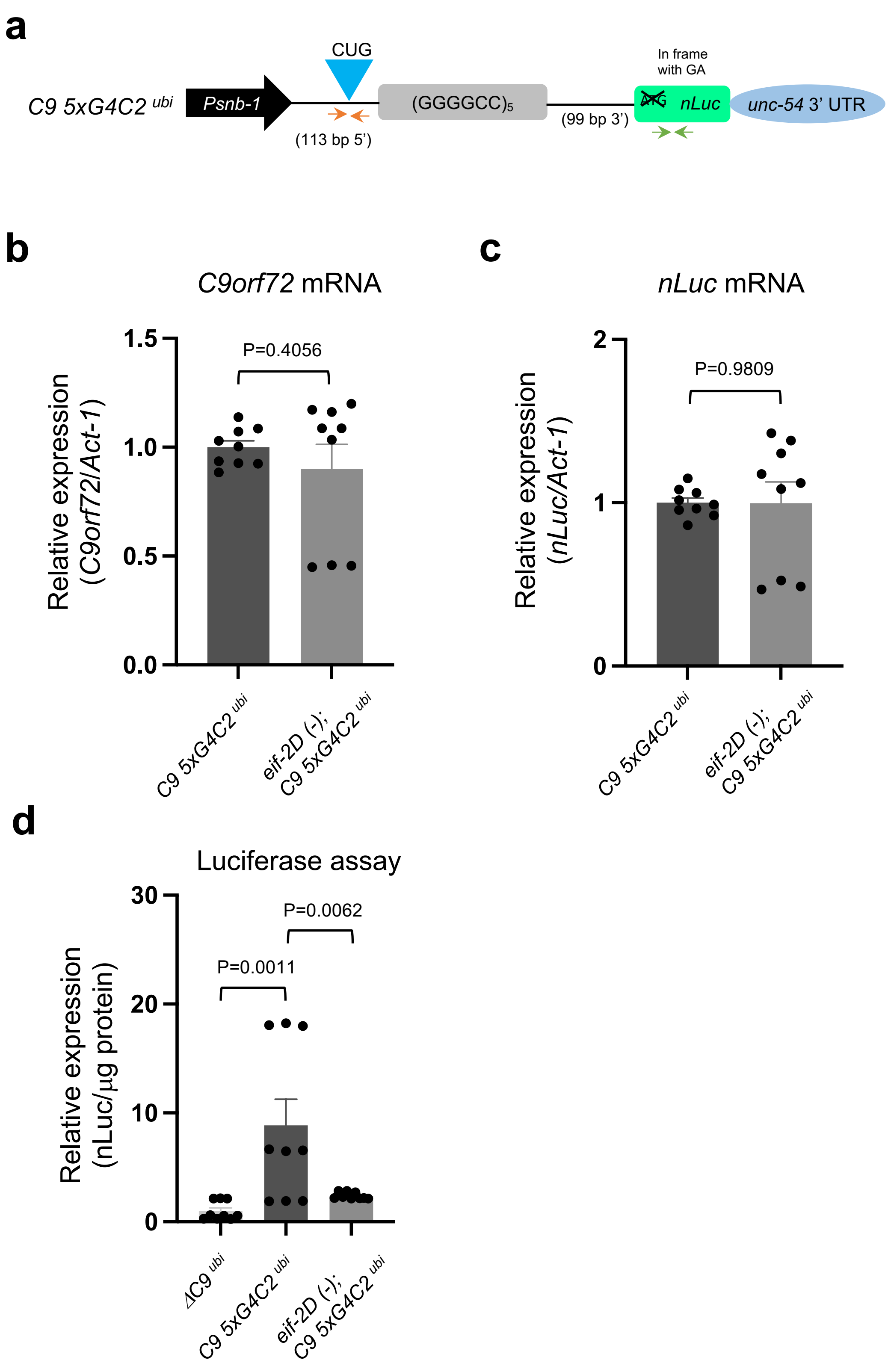

Supplementary figure 23. Knockout of *eif-2D* does not affect the levels of *C9orf72* intronic RNA and *nLuc* mRNA in *C9 5xG4C2 ubi* animals. (a) Schematic showing the *C9 5xG4C2 ubi* construct. The orange and green arrows show the location of primers designed to detect *C9orf72* and nLuc mRNAs, respectively. nLuc: nanoluciferase; UTR: untranslated region. The *C9orf72* intronic RNA (b) and *nLuc* (c) mRNAs were assessed by RT-PCR and normalized to Act-1 mRNA. The experiments were repeated 3 times. (d) A luciferase assay was performed on worm lysates. The experiments were repeated 3 times. Mean ± s.e.m. Two-tailed unpaired t-test was performed in (b, c) and One-way ANOVA with Dunnett's multiple comparisons test was performed in (d). The *eif-2D (gk904876)* allele was used. Transgenic Lines used: *DC9 ubi (KasEx154), C9 5xG4C2 ubi (KasEx244).*

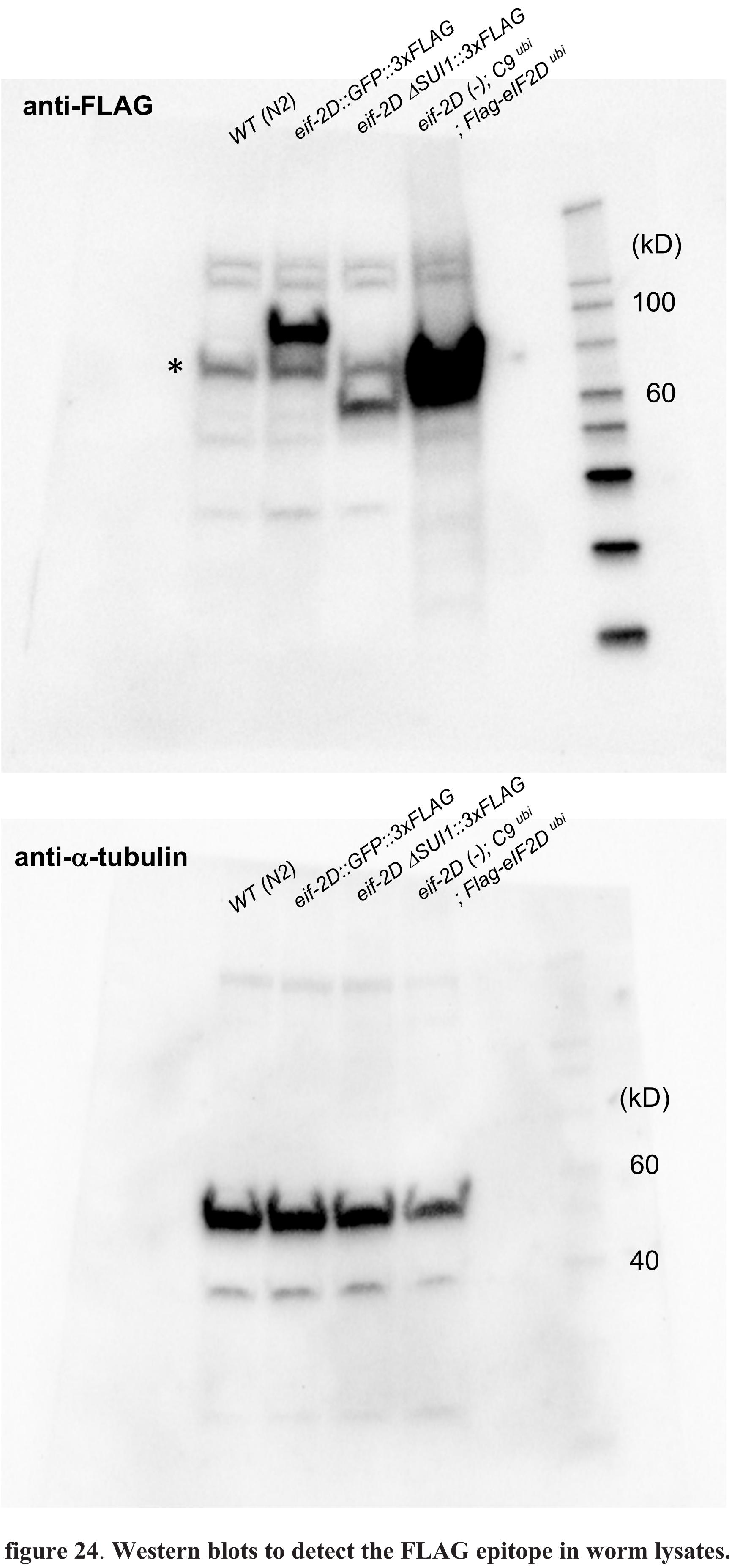

Supplementary figure 24. **Western blots to detect the FLAG epitope in worm lysates.** The lysates from WT (N2), *eif-2D::GFP::3xFLAG*, *eif-2 ΔSUI1::3xFLAG* and *eif-2D (gk904876)*; C9 ^{ubi}; *Flag-eIF2D* ^{ubi} *(KasEx248)* animals were processed for Western blotting and immunostained with Flag and α-tubulin antibodies. Asterisk shows non-specific band.

a

b

c

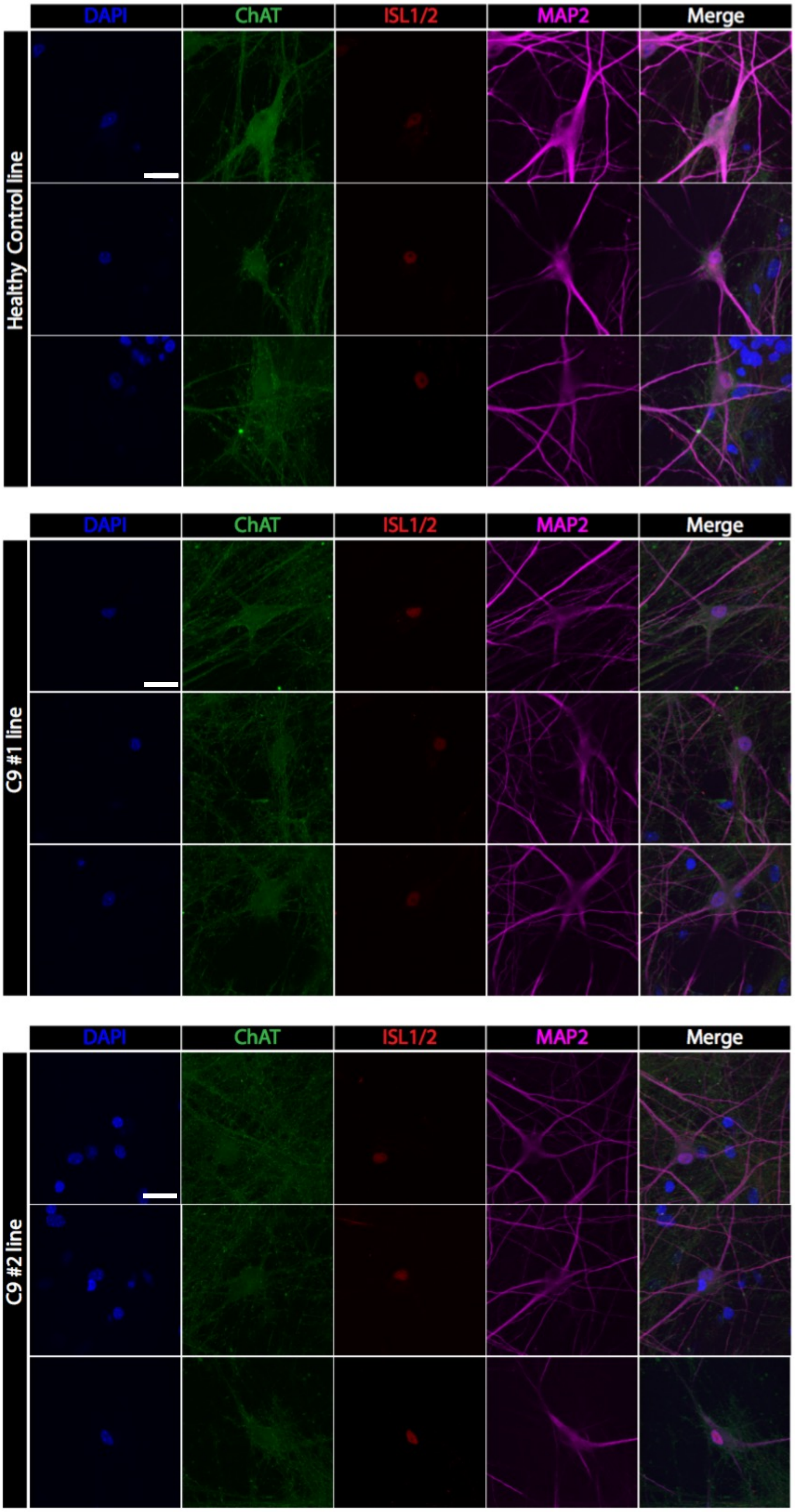

Supplementary figure 25. Human iPSC-derived motor neurons express neuronal markers ChAT, ISL1/2 and MAP2 at 50 days in culture. (a) Immunofluorescence staining against ChAT, ISL1/2, and MAP2. Three separate images of neurons derived from a healthy control iPSC line. Scale bar = 25 µm. (b) Three separate images of neurons derived from an iPSC line with C9ORF72 hexanucleotide expansion (C9#1 line). (c) Three separate images of neurons derived from an iPSC line with C9ORF72 hexanucleotide expansion (C9#2 line).

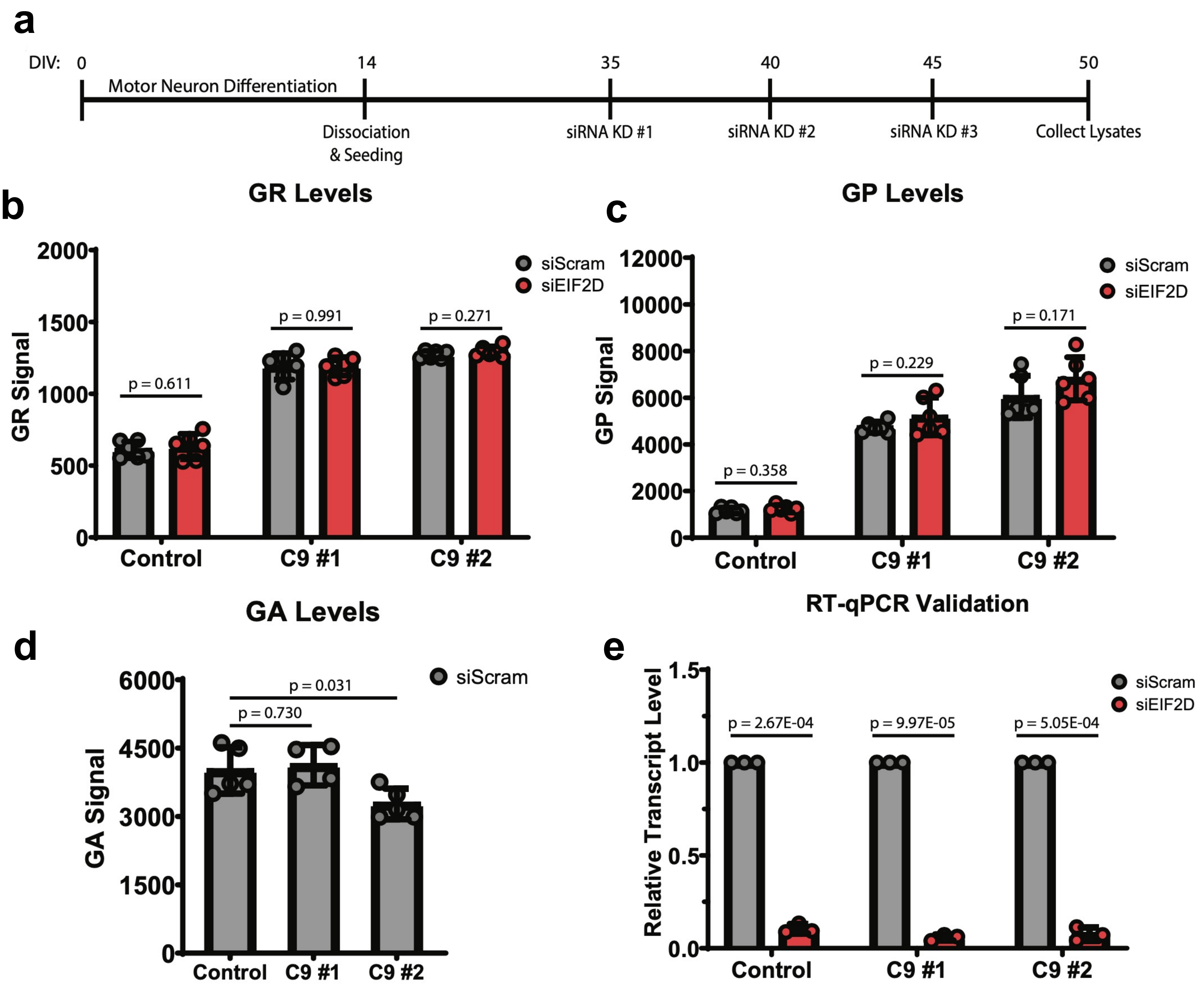

Supplementary figure 26. Kockdown of eIF2D does not alter the levels of poly-GP and poly-GR in iPSC-derived motor neurons. (a) Schematic of experimental design. D0-D15: small molecule driven lower motor neuron diffentiation. D35, D40, D45: siRNA knockdown of EIF2D. D50: collect material for analysis. DIV = days in vitro. (b-d) The lysates from iPSC-derived motor neurons were processed for poly-GR(b), poly-GP(c), and poly-GA(d) ELISA. (e) The EIF2D, GAPDH, and Actin mRNA levels were assessed by RT-PCR. The EIF2D transcript levels were normalized to GAPDH/Actin transcript levels. The experiments were repeated 3 times. Each dot represents individual replicates within each experiment; mean ± s.e.m. Two-tailed unpaired t-test was performed. siRNA: small interfering RNA; KD: knockdown; siScram: siScramble.

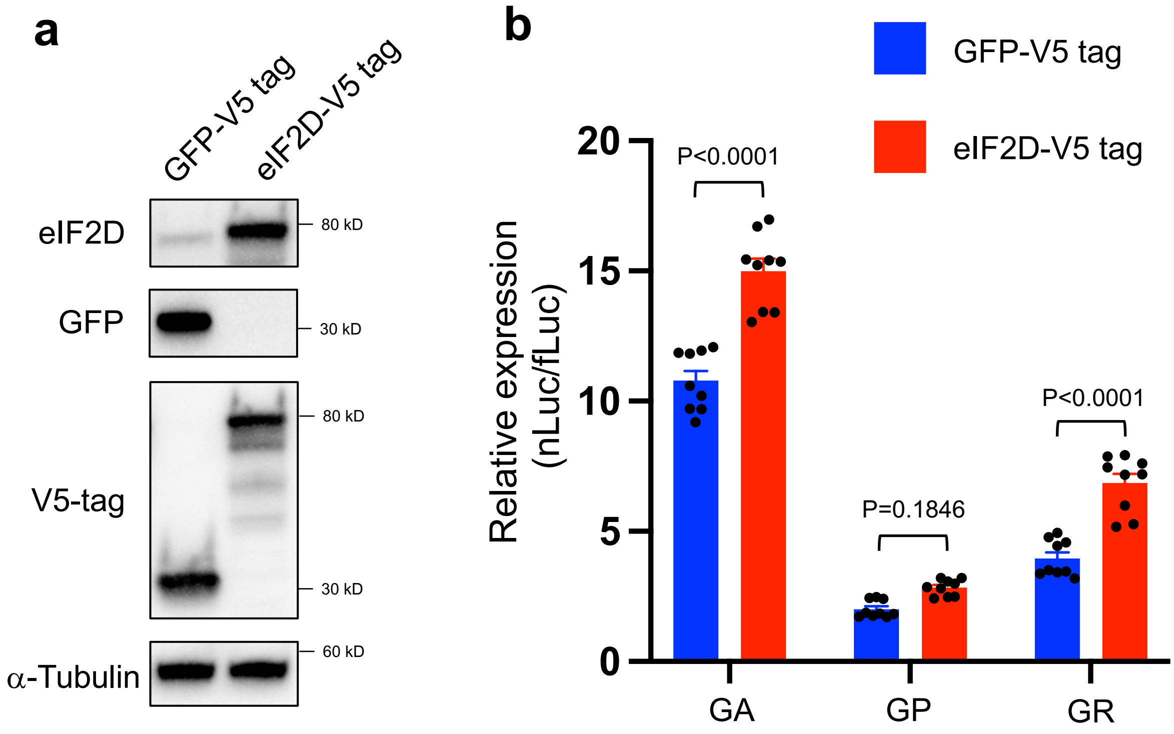

Supplementary figure 27. Overexpression of eIF2D enhances DPR production in HEK293 cells. Bicistronic constructs carrying 75 copies of the G4C2 repeat and flanking intronic sequences were cotransfected with constructs expressing either GFP (tagged with V5 epitope) or eIF2D (tagged with V5 epitope) and cultured for 48h. (a) Cell lysates were processed for Western blotting, and immunostained with antibodies to eIF2D, GFP, V5-tag, and α-tubulin. (b) The levels of luciferase activity were assessed by dual luciferase assays. The experiments were repeated 3 times. mean ± s.e.m. Two-way ANOVA with Šídák's multiple comparison test was performed.

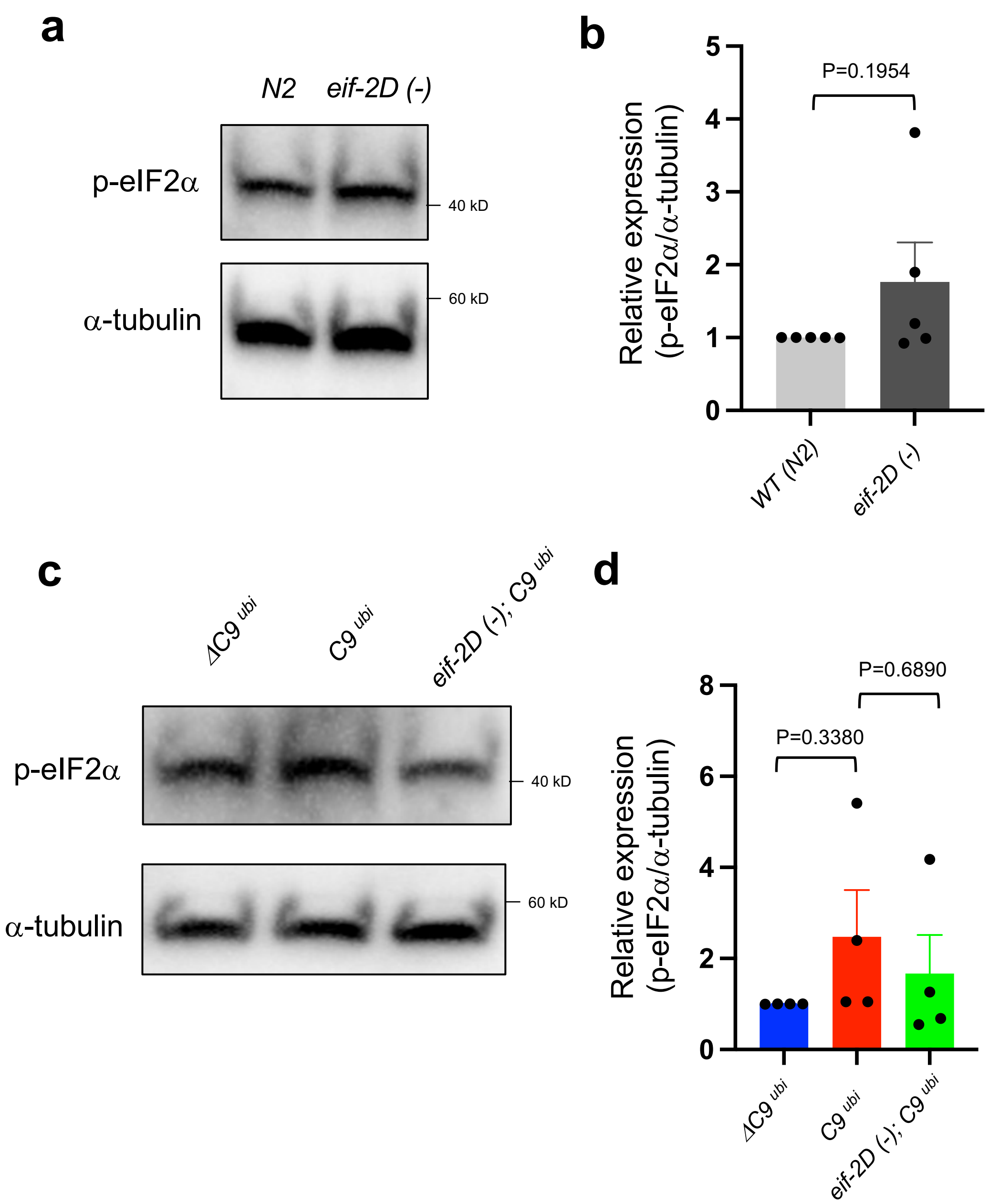

Supplementary figure 28. Knockout of *eif-2D* does not affect the levels of p-eIF2α in *C. elegans*. Lysates were processed for Western blotting from N2 wild type, *eif-2D (gk904876), ΔC9 ubi (KasEx154), C9 ubi (KasEx153),* and *eif-2D (-);* C9 *ubi* worms. A representative blot in (a) and (c) was immunostained with p-eIF2α and α-tubulin antibodies. Quantification is shown respectively in (b) and (d). The experiments were repeated 5 times in b and 4 times in d. Mean ± s.e.m. Two-tailed unpaired t-test was performed in (b) and One-way ANOVA with Dunnett's multiple comparisons test was performed in (d).

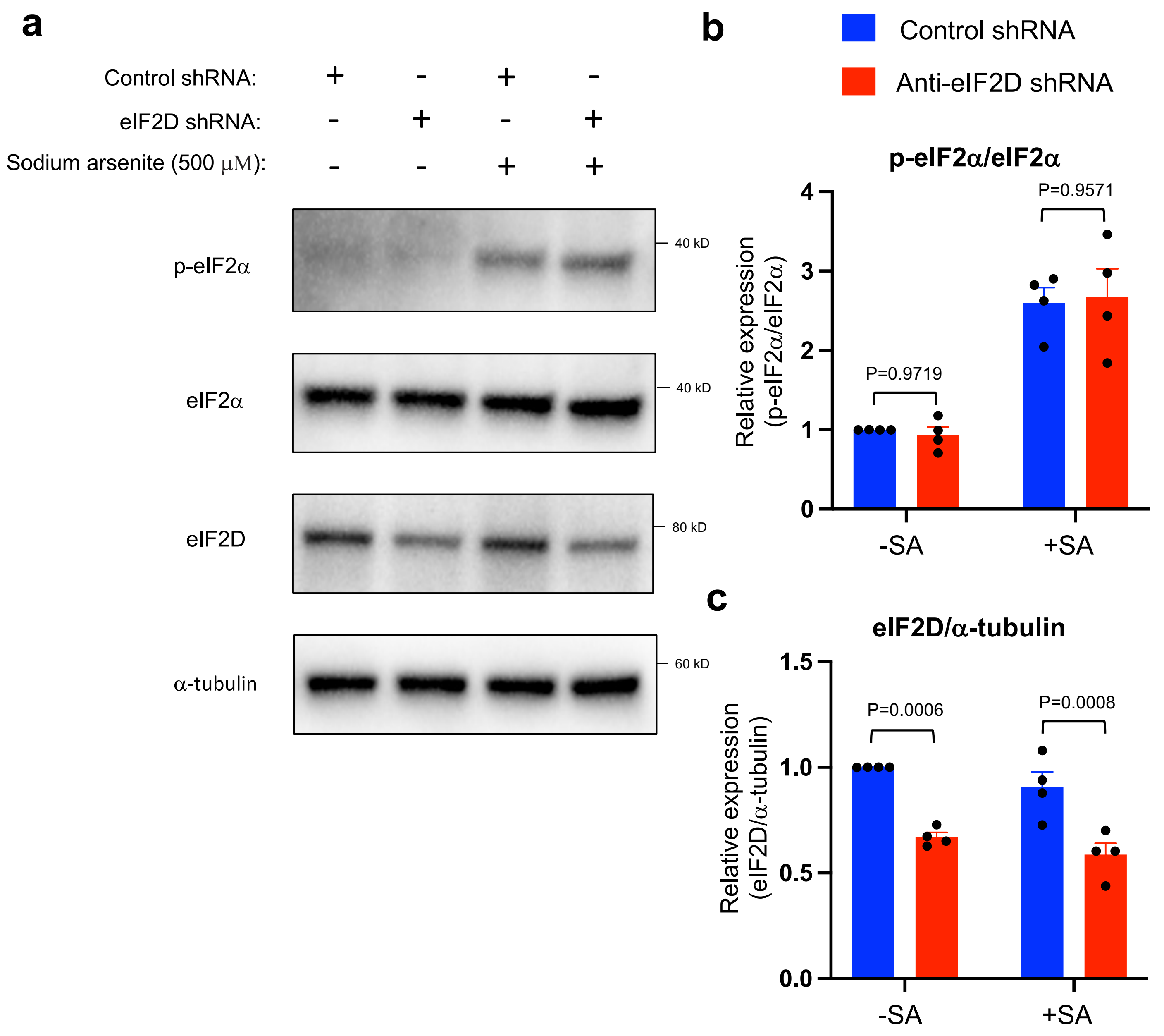

Supplementary figure 29. Knockdown of eIF2D does not affect the levels of p-eIF2α in HEK293 cells. HEK293 cells were transfected with control shRNA or eIF2D shRNA. The cells were treated 48h later with 500 μM sodium arsenite (SA), and after 30 min the cell lysates were processed for Western blotting. (a) A representative western blot immunostained with p-eIF2α, eIF2α, eIF2D, and α-tubulin. Quantification is provided for p-eIF2α/eIF2α (b) and eIF2D/α-tubulin (c). The experiments were repeated 4 times. mean ± s.e.m. Two-way ANOVA with Šídák's multiple comparison test was performed.

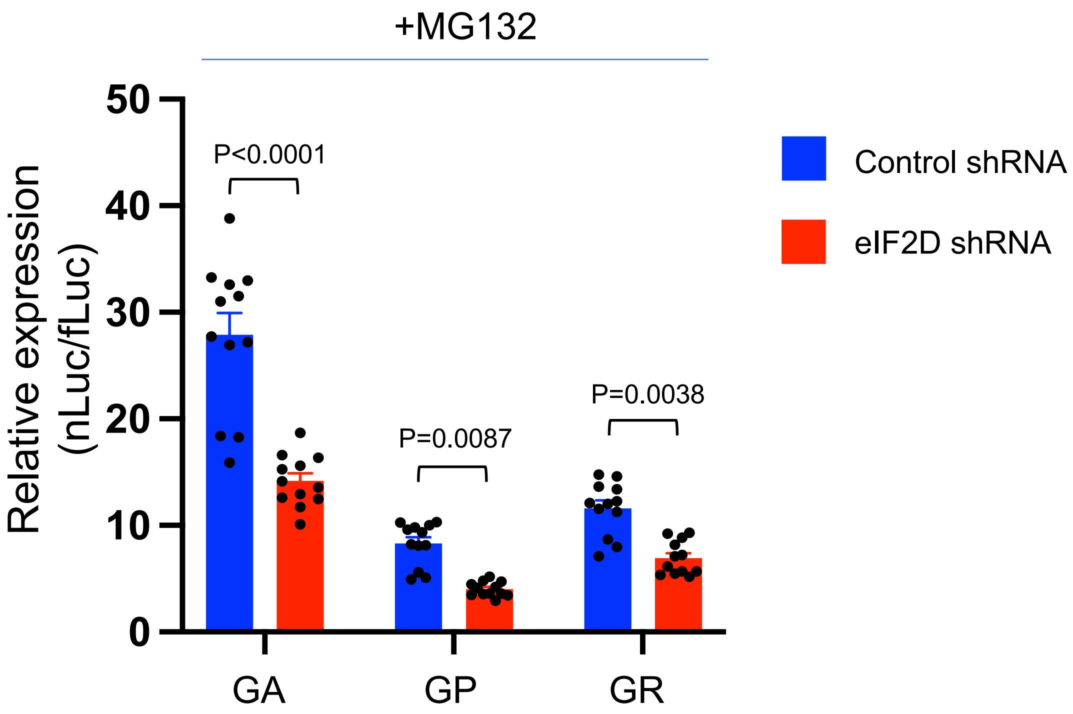

Supplementary figure 30. Treatment with a proteasome inhibitor (MG132) does not influence the effect of eIF2D knockdown on DPR production. Bicistronic constructs carrying 75 copies of the G4C2 repeat and flanking intronic sequences were cotransfected with constructs expressing either control shRNA or eIF2D shRNA and cultured for 48h. For the last 6h, 10 µM MG132 was added to the culture. The levels of luciferase activity were assessed by dual luciferase assays. The experiments were repeated 4 times, mean ± s.e.m. Two-way ANOVA with Šídák's multiple comparison test was performed.

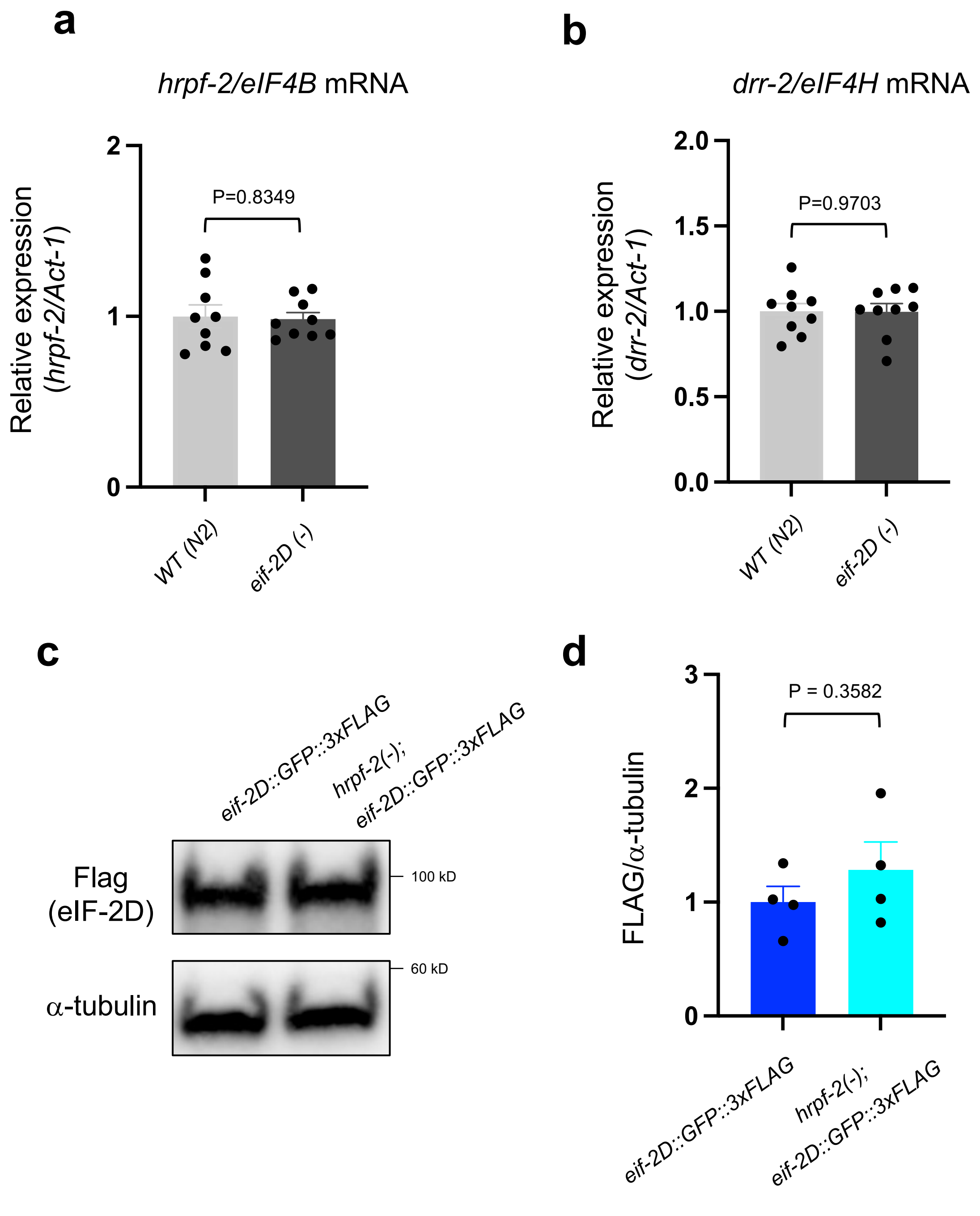

Supplementary figure 31. The translation initiation factors *eif-2D/eIF2D, hrpf-2/eIF4B*, and *drr-2/eIF4H* do not cross-regulate their expression. (a, b) Total RNA was extracted from N2 wild type and *eif-2D (gk904876)* animals. The *hrpf-2* (ortholog of human eIF4B) and *drr-2* (ortholog of human eIF4H) mRNAs levels were assessed by RT-PCR and normalized to *act-1*. The experiments were repeated 3 times. Two-tailed unpaired t-test was performed. (c, d) Lysates were processed for Western blotting from animals carrying the *eif-2D::GFP::3xFLAG* allele and *hrpf-2 (*gk5105*)* mutants carrying the same allele. A representative blot was immunostained with Flag and α-tubulin antibodies. (d) Quantification of panel c. The experiments were repeated 4 times. The data show mean ± s.e.m. Two-tailed unpaired t-test was performed.

Supplementary Table 1. List of all primers used in this study.

**Name**	**Purpose**	**Sequence (5'->3')**
Ce-eIF2A-F	Genotyping	aaggaaaaaagctagctgggcagtttacggaccaaa
Ce-eIF2A-R	Genotyping	aaggaaaaaacttaaggaagttggtcgcacccttct
Ce-eIF2D (gk904876)-F	Genotyping	aaggaaaaaagctagcaccgtttacagtcaagaaaaataca
Ce-eIF2D (gk904876)-R	Genotyping	aaggaaaaaacttaaggtgtagacattcatctgggttcct
Ce-eIF2D (gk561128)-F	Genotyping	acataagatggcctgacggc
Ce-eIF2D (gk561128)-R	Genotyping	ggcactgataattcaccaagcg
Ce-eif-2D-SUI>Flag-F	Genotyping	ggctgctcaaaaggggatct
Ce-eif-2D-SUI>Flag-R	Genotyping	cccatacttagaaacacacggg
Ce-pSnb-1-F	Cloning	aaacccactagtcagttcgggtatctcagcaa
Ce-pSnb-1-R	Cloning	acagggaagcttgtcgtcaagatggtcttatc
Ce-pUnc11c-F	Cloning	aaacccactagtctgtgtctctccgtcctatc
Ce-pUnc11c-R	Cloning	aaacccaagcttccaaataaaaggagctgtgt
Ce-Unc54-F	Cloning	aaacccctcgaggcgccggtcgctaccattac
Ce-Unc54-R	Cloning	acaggggagctcggaaacagttatgtttggta
Ce-eif2D-F	Cloning	aaacccgcggccgcattcaaaaaaccgtttacagt
Ce-eif2D-R	Cloning	aaacccggatccttatttcttctttttcaccg
Hs-eIF2D-F	Cloning	aaacccgaattcgccatgtttgccaaggcctttcg
Hs-eIF2D-R	Cloning	aaacccgcggccgctgcttcttcttgccaggtttga
GFP-F	Cloning	aaacccgaattcaccatggtgagcaagggcgagga
GFP-R	Cloning	cccaaagcggccgctgcttgtacagctcgtccatgc
GFP-novo2-F	Cloning	aaacccgcggccgcaagtaaaggagaagaactttt
GFP-novo2-R	Cloning	aaacccggatccctatttgtatagttcatcca
∆G4C2-1-F	Cloning	agcttagtactcgctgagggtgaacaagaaaagacctgataaagattaaccagaagaaaacaaggagggaaacaaccgcagcctgtagcaagctctggaactcag
∆G4C2-1-R	Cloning	gttccagagcttgctacaggctgcggttgtttccctccttgttttcttctggttaatctttatcaggtcttttcttgttcaccctcagcgagtacta
∆G4C2-2-F	Cloning	gagtcgcgcgctaggggcgtggtcggggcgggcccggg ggcgggcccggggcggggctgcggttgcggtgcctgcgcccgcggcggcggaggcgcaggcggtggcgagtggggg atcaaacgc
∆G4C2-2-R	Cloning	ggccgcgtttgatcccccactcgccaccgcctgcgcctccgccgccgcgggcgcaggcaccgcaaccgcagccccgccccgggcccgcccccgggcccgccccgaccacgcccctagcgcgcgactcctga
∆nLuc-F	Cloning	ggccgcgatcctagttaagtagcg
∆nLuc-R	Cloning	gatccgctacttaactaggatcgc
3xFlag-F	Cloning	agcttatggattataaagacgatgacgataagcgtgactacaaggacgacgacgacaagcgtgattacaaggatgacgatgacaagagagc
3xFlag-R	Cloning	ggccgctctcttgtcatcgtcatccttgtaatcacgcttgtcgtc

		gtcgtccttgtagtcacgcttatcgtcatcgtctttataatccata
Hs-eIF2D-shRNA-F	Cloning	gatccgggacgacaactggacataaagttcaagagactttatgtccagttgtcgtcctttttggaaa
Hs-eIF2D-shRNA-R	Cloning	agcttttccaaaaaggacgacaactggacataaagtctcttgaactttatgtccagttgtcgtcccg
Mm-eIF2D-shRNA-1-F	Cloning	ccggcgccgaaagatcatcacggactactcgagtagtccgtgatgatctttcggtttttg
Mm-eIF2D-shRNA-1-R	Cloning	aattcaaaaaccgaaagatcatcacggactactcgagtagtccgtgatgatctttcggcg
Mm-eIF2D-shRNA-2-F	Cloning	ccggcgcccattctaaagaaagggaaactcgagtttccctttctttagaatgggtttttg
Mm-eIF2D-shRNA-2-R	Cloning	aattcaaaaacccattctaaagaaagggaaactcgagtttccctttctttagaatgggcg
Mm-eIF2D-shRNA-3-F	Cloning	ccggcgcctgcctaccaagtaacatttctcgagaaatgttacttggtaggcaggtttttg
Mm-eIF2D-shRNA-3-R	Cloning	aattcaaaaacctgcctaccaagtaacatttctcgagaaatgttacttggtaggcaggcg
Ce-qPCR-Act-1-F	qPCR	acgacgagtccggcccatcc
Ce-qPCR-Act-1-R	qPCR	gaaagctggtggtgacgatggtt
qPCR-C9-F	qPCR	gaaacaaccgcagcctgtag
qPCR-C9-R	qPCR	tagcgcgcgactcctga
qPCR-nLuc-F	qPCR	cagccggctacaacctggac
qPCR-nLuc-R	qPCR	agcccattttcaccgctcag
Ce-qPCR-hrpf2-F	qPCR	gcagtgaaaactcaatccgca
Ce-qPCR-hrpf2-R	qPCR	ggcttcttggtggttctcca
Ce-qPCR-drr2-F	qPCR	tcagtggccttaatggagcc
Ce-qPCRdrr2-R	qPCR	gaagtttcctccgcgtcctc
Hs-qPCR-EIF2D-F	qPCR	cggacaggaagcttcga
Hs-qPCR-EIF2D-R	qPCR	ccaccactcacgtacacagt
Hs-qPCR-ACTIN-F	qPCR	tttgagaccttcaacaccccagcc
Hs-qPCR-ACTIN-R	qPCR	aatgtcacgcacgatttcccgc
Hs-qPCR-GAPDH-F	qPCR	gtctacatggcaactgtgagga
Hs-qPCR-GAPDH-R	qPCR	gatgacatcaagaaggtggtga

Supplementary File 1. Pluripotency certificate for ALS patient iPSC line C9 #1 (TALS9-11.2) obtained from Target ALS. Cat# ND50008; RRID:CVCL_FA04. Female, 61 years old. See next page.

RUCDR INFINITE BIOLOGICS

Date - 03/03/2017

NHCDR ID - ND50008
Cell line ID - FA0000018
Lot numbers -R013356330
R013535970
Depositor - Target ALS

Source cell type - FCL
Reprogramming method - Sendai viral vectors
Growth conditions - mTeSR and Matrigel (SOP# G103-0179)
Passage method - Dispase (SOP# G103-0179)
Total Passage Number - 17
Number of passages at RUCDR - 10
Number of cells /vial -2.0 x 10^6

Property	Test Name	Test Method	Criteria for Passing	Test Result
**Sterility**	Mycoplasma Contamination	qPCR	Negative	Pass
Identity	SNP Trace	96 SNP assay	Matches source cells, if available	Pass
Pluripotency	Live colony image	Visual inspection	Majority of colonies have smooth edges, are mostly circular and consist of small, tightly compacted cells with large nuclear to cytoplasm ratios	Pass
	AP Stain	Live cell fluorescent stain for Alkaline Phosphotase	Posititve	Pass
	Immunoflu-orscence	Fixed cell staining for Oct4 and Tra-1-60	Expression of Oct4 and Tra-1-60	N/A
	FACS	Flow cytometry for the expres-sion of Oct4 and Tra-1-60	Minimum 90% Oct4/Tra-1-60 double positive	95%-97%
	RUCDR Pluritest	HT12 array for gene expression	>18 Pluripotency	N/A
			<2.1Novelty	N/A
	iPSC Scorecard	96 well qPCR assy on undifferentiated cells for the expression of pluripotency markers	Positive score for pluripotency and negative score for differentiation	N/A
	Differentiation Scorecard	96 well qPCR assay on differentiated cells for the expression of markers of all 3 germ layers	Negative score for pluripotency and positive score for differentiation	N/A
Exogenous repro-gramming factor expression	Sendai Persistence	qPCR assay to dectect sendai viral sequences	< 1.0 RNA molecules per cell	N/A
			R^2 value ≥ 0.95	N/A
	Episome Presistence	qPCR assay to dectect episome sequences	< 0.5 DNA molecules per cell	N/A
			R^2 value ≥ 0.95	N/A
Genetic Stability	Karyotyping	G-band analysis	Expected chromosome complement (Dependant on subject)	N/A
Viability	Test Thaw	Live cell dye followed by con-fluency quantitation	At least 10 pluripotent colonies at 72hr post-thaw	Pass

Cryopreservation

Cryopreservation date -01/04/2016
Cryopreservation method - accutase and mFreSR (SOP# G103-0179)
Cell density - 2.0x 10^6 /vial

	R013356330	R013535970
Recomended thaw density	1 vial in 1 well(s) of a 6 well plate (9.6cm^2)	1 vial in 1 well(s) of a 6 well plate (9.6cm^2)
# Colonies after 24 hours	>10	>10
# Colonies after 72 hours	>30	>30

Post thaw viability

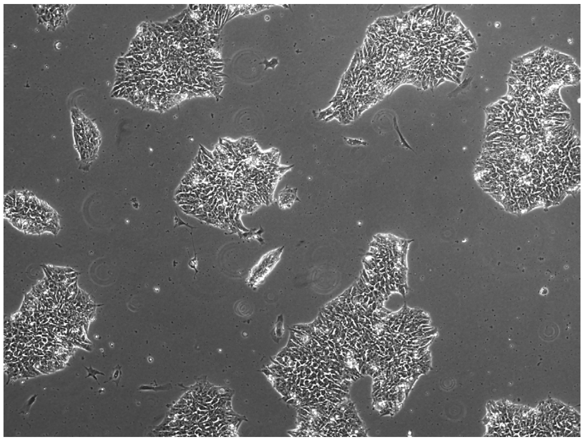

10x magnification 3 days after (R013356330)

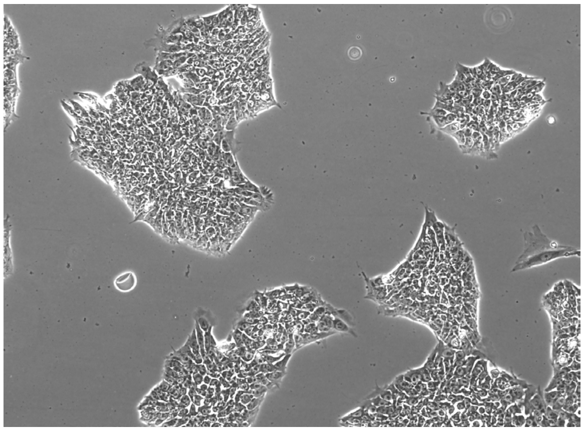

10x magnification 3 days after thaw (R013535970)

Pluripotency

Live Colony Image (10x)

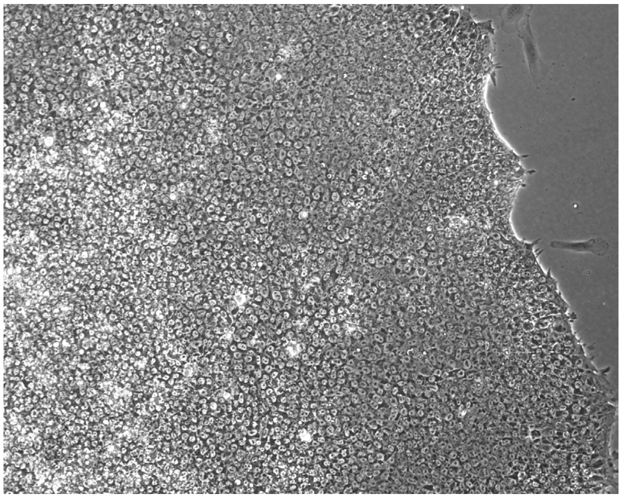

AP stain (10X)

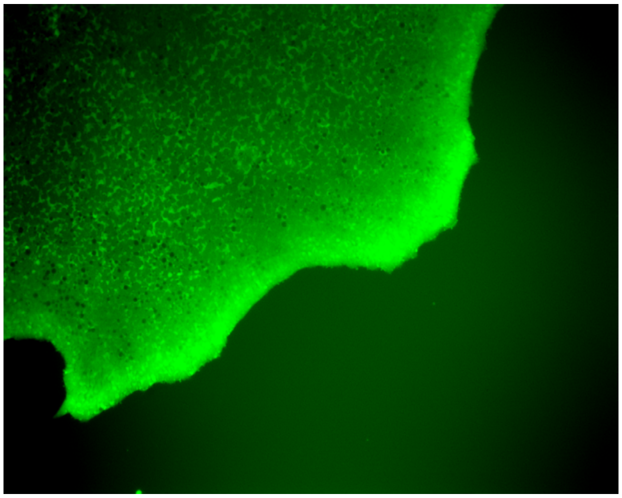

FACS Analysis

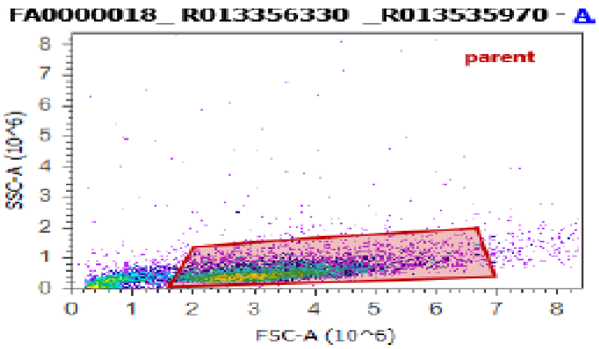

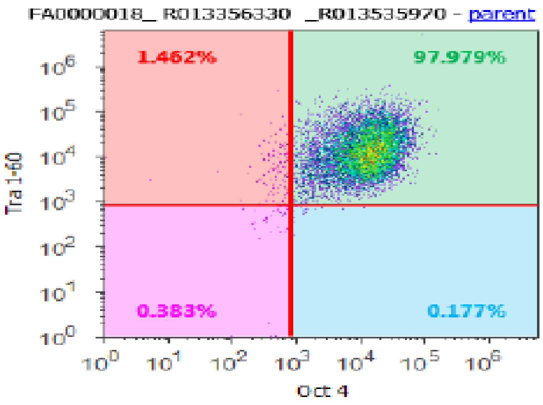

Supplement: Supplementary file 1 — Supplementary Information [file 41467_2021_26303_MOESM1_ESM.pdf]
