# Supplementary material for: A C. elegans model of C9orf72-associated ALS/FTD uncovers a conserved role for eIF2D in RAN translation
Source: Nat Commun. 2021 Oct 15;12:6025. doi: 10.1038/s41467-021-26303-x (PMC8519953; doi:10.1038/s41467-021-26303-x)
Supplement: Supplementary file 9 — Description of additional supplementary files [file 41467_2021_26303_MOESM9_ESM.docx]

Description of additional supplementary files

Title: Supplementary Movie 1

Description: Freely moving adult (day 6) ΔC9 ubi transgenic animal.

Title: Supplementary Movie 2

Description: Freely moving adult (day 6) C9 ubi transgenic animal.

Title: Supplementary Movie 3

Description: Freely moving adult (day 6) UAG ubi transgenic animal.

Title: Supplementary Data 1

Description: Promoter sequences used for C. elegans transgenic animals.

Title: Supplementary Data 2

Description: List of plasmids generated for this study.

Title: Supplementary Data 3

Description: C. elegans strains used in this study.
