# Supplementary material for: A C. elegans model of C9orf72-associated ALS/FTD uncovers a conserved role for eIF2D in RAN translation
Source: Nat Commun. 2021 Oct 15;12:6025. doi: 10.1038/s41467-021-26303-x (PMC8519953; doi:10.1038/s41467-021-26303-x)
Supplement: Supplementary file 10 — Reporting Summary [file 41467_2021_26303_MOESM10_ESM.pdf]

nature research

Corresponding author(s): Raymond P. Roos, Paschalis Kratsios

Last updated by author(s): Sep 12, 2021

Reporting Summary

Nature Research wishes to improve the reproducibility of the work that we publish. This form provides structure for consistency and transparency in reporting. For further information on Nature Research policies, see Authors & Referees and the Editorial Policy Checklist.

Statistics

For all statistical analyses, confirm that the following items are present in the figure legend, table legend, main text, or Methods section.

n/a	Confirmed	
☐	☒	The exact sample size (*n*) for each experimental group/condition, given as a discrete number and unit of measurement
☐	☒	A statement on whether measurements were taken from distinct samples or whether the same sample was measured repeatedly
☐	☒	The statistical test(s) used AND whether they are one- or two-sided<br>*Only common tests should be described solely by name; describe more complex techniques in the Methods section.*
☒	☐	A description of all covariates tested
☐	☒	A description of any assumptions or corrections, such as tests of normality and adjustment for multiple comparisons
☐	☒	A full description of the statistical parameters including central tendency (e.g. means) or other basic estimates (e.g. regression coefficient) AND variation (e.g. standard deviation) or associated estimates of uncertainty (e.g. confidence intervals)
☐	☒	For null hypothesis testing, the test statistic (e.g. *F*, *t*, *r*) with confidence intervals, effect sizes, degrees of freedom and *P* value noted<br>*Give P values as exact values whenever suitable.*
☒	☐	For Bayesian analysis, information on the choice of priors and Markov chain Monte Carlo settings
☒	☐	For hierarchical and complex designs, identification of the appropriate level for tests and full reporting of outcomes
☒	☐	Estimates of effect sizes (e.g. Cohen's *d*, Pearson's *r*), indicating how they were calculated

Our web collection on statistics for biologists contains articles on many of the points above.

Software and code

Policy information about availability of computer code

Data collection	Image data for Western blotting were acquired using ChemiDoc MP Imaging System (BIO-RAD) and Image Lab software 6.0.1. (BIO-RAD). Luminescence was acquired using VICTOR3 1420 MULTILABEL COUNTER (PerkinElmer) and 2030 workstation software (version 4.0, PerkinElmer). The data for qPCR were acquired using CFX96 Real-Time System (BIO-RAD) and CFX Manager software (version 3.1, BIO-RAD). Image data for immunocytochemistry was acquired using Axio Imager Z2 (Zeiss) and ZEN software (Zeiss, Version 2.3.69.1000, Blue edition). The image reconstruction was performed using Image J software (version version 2.0.0-rc-59/1.51k). Locomotion analysis of videos of C. elegans animals was performed using Tierpsy Tracker (version 228).
Data analysis	Statistical analysis was performed by GraphPad Prism software version 8.2.1.

For manuscripts utilizing custom algorithms or software that are central to the research but not yet described in published literature, software must be made available to editors/reviewers. We strongly encourage code deposition in a community repository (e.g. GitHub). See the Nature Research guidelines for submitting code & software for further information.

Data

Policy information about availability of data

All manuscripts must include a data availability statement. This statement should provide the following information, where applicable:

- Accession codes, unique identifiers, or web links for publicly available datasets
- A list of figures that have associated raw data
- A description of any restrictions on data availability

The authors declare that all data supporting the findings of this study are available within the paper and its supplementary information files. Source data are provided with this paper.

Field-specific reporting

Please select the one below that is the best fit for your research. If you are not sure, read the appropriate sections before making your selection.

☒ Life sciences ☐ Behavioural & social sciences ☐ Ecological, evolutionary & environmental sciences

For a reference copy of the document with all sections, see nature.com/documents/nr-reporting-summary-flat.pdf

Life sciences study design

All studies must disclose on these points even when the disclosure is negative.

Sample size	We determined the sample size based on similar experimental setups described in previous publications [Nat Commun 8, 2005 (2017); eLife 9, e50065 (2020)]. Based on these studies, the sample size used in the current study is sufficient to find statistical significance.
Data exclusions	No data were excluded from this study.
Replication	All attempts at replication were successful. The experiments were replicated 3-5 times.
Randomization	The samples were randomly chosen and allocated into experimental groups. For experiments involving C. elegans, animals from each transgenic line were randomly selected.
Blinding	ELISA and locomotor assays were performed by investigators blinded to the experimental condition. For the remaining experiments, the investigators were not blinded to group allocation during data collection and analysis because the results are quantitative and do not require subjective interpretation.

Reporting for specific materials, systems and methods

We require information from authors about some types of materials, experimental systems and methods used in many studies. Here, indicate whether each material, system or method listed is relevant to your study. If you are not sure if a list item applies to your research, read the appropriate section before selecting a response.

Materials & experimental systems

n/a	Involved in the study	
☐	☒	Antibodies
☐	☒	Eukaryotic cell lines
☒	☐	Palaeontology
☐	☒	Animals and other organisms
☒	☐	Human research participants
☒	☐	Clinical data

Methods

n/a	Involved in the study	
☒	☐	ChIP-seq
☒	☐	Flow cytometry
☒	☐	MRI-based neuroimaging

Antibodies

Antibodies used

Mouse anti-poly-GA monoclonal antibody (EMD Millipore) Catalog#: MABN889, clone 5E9;
Goat anti-firefly luciferase polyclonal antibody (Promega) Catalog# G7451;
Rabbit anti-FLAG polyclonal antibody (Sigma) Catalog# F7425;
Mouse anti-DYKDDDDK Tag monoclonal antibody (Cell Signaling Technology) Catalog# 8146, clone 9A3;
Rabbit anti-GFP monoclonal antibody (Cell Signaling Technology) Catalog# 2956, clone D5.1;
Rabbit anti-V5-Tag monoclonal antibody (Cell Signaling Technology) Catalog# 13202, clone D3H8Q;
Rabbit anti-HA-Tag monoclonal antibody (Cell Signaling Technology) Catalog# 3724, clone C29F4;
Rabbit anti-eIF2D polyclonal antibody (Abcam) Catalog# ab108218;
Rabbit anti-LGTN polyclonal antibody (Proteintech) Catalog# 12840-1-AP;
Rabbit anti-Phospho-eIF2α (Ser51) monoclonal antibody (Cell Signaling Technology) Catalog# 3597, clone 119A11;
Mouse anti-eIF2α monoclonal antibody (Cell Signaling Technology) Catalog# 2103, clone L57A5;
Goat anti-ChAT polyclonal antibody (Millipore) Catalog# AB144P;
Mouse anti-ISLET 1/2 monoclonal antibody (DSHB) Catalog# 39.4D5, clone;
Chicken anti-MAP2 polyclonal antibody (Abcam) catalog# ab5392;
Rat anti-tubulin monoclonal antibody (Abcam) Catalog# ab6160, clone YL1/2;
Mouse anti-β-actin monoclonal antibody (Sigma) Catalog# A5441, clone AC-15;
Sheep HRP-conjugated anti-mouse IgG secondary antibody (GE Healthcare) Catalog# NA931V
Donkey HRP-conjugated anti-rabbit IgG secondary antibody (GE Healthcare) Catalog# NA9340V
Goat HRP-conjugated anti-rat IgG secondary antibody (Cell Signaling Technology) Catalog# 7077S;
Donkey Alexa 488-conjugated anti-goat IgG (H+L) secondary antibody (Thermo Fisher Scientific) Catalog# A11055
Donkey Alexa 555-conjugated anti-mouse IgG (H+L) secondary antibody (Thermo Fisher Scientific) Catalog# A31570

	Donkey Alexa 647-conjugated anti-chicken IgG (H+L) secondary antibody (Jackson ImmunoResearch) Catalog# 703-605-155<br>Goat Alexa 594-conjugated anti-mouse IgG (H+L) secondary antibody (Thermo Fisher Scientific) Catalog# A11020
Validation	The antibodies used in this study were validated by the manufacturers as shown in the following websites.<br>Anti-poly-GA: https://www.emdmillipore.com/US/en/product/Anti-C9ORF72-C9RANT-poly-GA-Antibody-clone-5E9,MM_NF-MABN889<br>Anti-firefly luciferase: https://www.promega.com/products/protein-detection/primary-and-secondary-antibodies/anti-luciferase-pab/?catNum=G7451<br>Anti-FLAG: https://www.sigmaaldrich.com/US/en/product/sigma/f7425<br>Anti-DYKDDDDK Tag: https://www.cellsignal.com/products/primary-antibodies/dykddddk-tag-9a3-mouse-mab-binds-to-same-epitope-as-sigma-s-anti-flag-m2-antibody/8146<br>Anti-GFP: https://www.cellsignal.com/products/primary-antibodies/gfp-d5-1-rabbit-mab/2956<br>Anti-V5 Tag: https://www.cellsignal.com/products/primary-antibodies/v5-tag-d3h8q-rabbit-mab/13202<br>Anti-HA Tag: https://www.cellsignal.com/products/primary-antibodies/ha-tag-c29f4-rabbit-mab/3724<br>Anti-eIF2D: https://www.abcam.com/eif2d-antibody-ab108218.html<br>Anti-LGTN: https://www.ptglab.com/products/LGTN-Antibody-12840-1-AP.htm<br>Anti-anti-Phospho-eIF2α (Ser51): https://www.cellsignal.com/products/primary-antibodies/phospho-eif2a-ser51-119a11-rabbit-mab/3597<br>Anti-eIF2α: https://www.cellsignal.com/products/primary-antibodies/eif2a-l57a5-mouse-mab/2103<br>Anti-ChAT: https://www.emdmillipore.com/US/en/product/Anti-Choline-Acetyltransferase-Antibody,MM_NF-AB144P<br>Anti-ISLET 1/2: https://dshb.biology.uiowa.edu/39-4D5<br>Anti-MAP2: https://www.abcam.com/map2-antibody-ab5392.html<br>Anti-tubulin: https://www.abcam.com/tubulin-antibody-yl12-loading-control-ab6160.html<br>Anti-β-actin: https://www.sigmaaldrich.com/US/en/product/sigma/a5441<br>HRP-anti-mouse IgG: https://cdn.cytivalifesciences.com/dmm3bwsv3/AssetStream.aspx?mediaformatid=10061&destinationid=10016&assetid=11297<br>HRP-anti-rabbit IgG: https://cdn.cytivalifesciences.com/dmm3bwsv3/AssetStream.aspx?mediaformatid=10061&destinationid=10016&assetid=11327<br>HRP-anti-rat IgG: https://www.cellsignal.com/products/secondary-antibodies/anti-rat-igg-hrp-linked-antibody/7077<br>Alexa 488-anti-goat IgG: https://www.thermofisher.com/antibody/product/Donkey-anti-Goat-IgG-H-L-Cross-Adsorbed-Secondary-Antibody-Polyclonal/A-11055<br>Alexa 555-anti-mouse IgG: https://www.thermofisher.com/antibody/product/Donkey-anti-Mouse-IgG-H-L-Highly-Cross-Adsorbed-Secondary-Antibody-Polyclonal/A-31570<br>Alexa 647-anti-chicken IgG (H+L): https://www.jacksonimmuno.com/catalog/products/703-605-155<br>Alexa 594-anti-mouse IgG: https://www.thermofisher.com/antibody/product/Donkey-anti-Mouse-IgG-H-L-Highly-Cross-Adsorbed-Secondary-Antibody-Polyclonal/A-31570

Eukaryotic cell lines

Policy information about cell lines

Cell line source(s)	The HEK293 cell line was obtained from ATCC (CRL-1573). The NSC34 cell line was a generous gift from Dr. Neil R. Cashman (McGill University, Montreal, QC, Canada). The NSC34 cell line is described in PMID: 1467557.
Authentication	None of the cell lines used were authenticated .
Mycoplasma contamination	The cell lines were not tested for mycoplasma contamination.
Commonly misidentified lines<br>(See ICLAC register)	No commonly misidentified cell lines were used.

Animals and other organisms

Policy information about studies involving animals; ARRIVE guidelines recommended for reporting animal research

Laboratory animals	The following C. elegans strains were used in this study at larval (stage 4) and adult (day 1 - 30) stages:<br>PHX3364, PHX3432, KRA314, KRA315, KRA316, KRA317, KRA522, KRA443, KRA551, KRA552, KRA444, KRA549, KRA550, KRA445, KRA553, KRA554, KRA448, KRA450, KRA451, KRA452, KRA524, KRA527, KRA531, KRA534, KRA540, KRA535, KRA543, KRA544, KRA545, VC20620, VC40968, VC40299, VC4032, LX929, OH7193
Wild animals	No wild animals were used in this study.
Field-collected samples	No field collected samples were used in the study.
Ethics oversight	The study did not require an ethical approval.

Note that full information on the approval of the study protocol must also be provided in the manuscript.
